# Supplementary material for: A Sensorised Glove to Detect Scratching for Patients with Atopic Dermatitis
Source: Sensors (Basel). 2023 Dec 12;23(24):9782. doi: 10.3390/s23249782 (PMC10748247; doi:10.3390/s23249782)
Supplement: Supplementary file 1 [file sensors-23-09782-s001.zip › sensors-2732441-supplementary.pdf]

Experiment Protocol

All actions are labelled according to this rule:

XYY

where X: 0 = Resting, 1 = Non-scratching, 2 = Scratching activities. YY is the type of action performed.

User is to start at resting position before each activity, and to return to resting position after each activity.

Operator will start recording, then instruct the user to perform the action. At around 7 secs into the recording, operator will instruct the user to return to resting position. Recording stops 1 second after user returns to resting position ***Rest (1 secs) –Movement (5secs) – Rest (1 secs)***

Part 0 Resting position

011. Subject sitting down on an immobile chair with a table in front. The user's forearm rests on the table with hanging elbows at 90 degrees, palm facing down and resting on the surface of the table – **10 secs**

Part 0 Closed grip

021. Subject in the resting position with the palm on the table, clenches the index, middle, ring and pinkie fingers as much as possible with a relaxed thumb ***"Thumbs up"*** – **5 secs**
022. Subject in the resting position, opposes the thumb as much as possible with the other fingers relaxed ***"Show 4"*** – – **5 secs**

Part 1 Non scratching activities

a. Handwaving
   111. Forearm hanging, waving the arm side-to-side, palm facing down – **10 secs**
   112. Forearm hanging, waving the arm side-to-side, palm facing medially – **10 secs**
   113. Forearm hanging, waving the arm side-to-side, palm facing forward – **10 secs**
   114. Forearm hanging, waving the arm forwards-and-back, palm facing forward – **10 secs**
   115. Forearm rested, waving palm facing down, side-to-side – **10 secs**
   116. Forearm rested, waving palm facing medially, side-to-side – **10 secs**
   117. Forearm rested, waving palm facing forward, side-to-side – **10 secs**
   118. Forearm rested, waving palm facing forward, forwards-and-back – **10 secs**

b. Typing
   121. User to type out provided message on his/her smartphone – **Until completion**
   122. User to type out provided message on a keyboard – **Until completion (~50s)**

```
A paralegal is a person trained in legal matters who performs tasks requiring knowledge of
the law and legal procedures. A paralegal is not a lawyer but can be employed by a law
office or work freelance at a company or law office.
```

c. Tapping on desk
   131. User to tap with palm resting on table, fingers extended – **10 secs**
   132. User to tap with palm resting on table, fingers bent – **10 secs**

d. Grab and Shake
   141. User to grab a can, and shake it up and down – **10 secs**
   142. User to grab a can, and shake it side to side – **10 secs**

e. Grab and Transfer
   151. User to grab a can, and transfer it over a partition – **Until completion**
   152. User to grab a small cube, and transfer it over a partition – **Until completion**

f. Turn the key
   161. User to turn the key until the lock is open – **Until completion**

g. Turn bottle cap
   171. User to separate the bottle and cap, place both on table – **Until completion**

172. User to fit cap onto the bottle, place bottle on table – **Until completion**

Part 2 Scratching/rubbing activities

*** User is to start at resting position before each activity, and to return to resting position after each activity.*

a. Back of Hand

211. Scratch by moving the whole forearm – **10 secs**
212. Scratch with fingers only – **10 secs**
213. Rubbing – **10 secs**
214. Scratch by moving the whole forearm (high) – **10 secs**
215. Scratch with fingers only (high) – **10 secs**
216. Rubbing (high) – **10 secs**

b. Forearm

221. Scratch by moving the whole forearm **– 10 secs**
222. Scratch with fingers only – **10 secs**
223. Rubbing – **10 secs**
224. Scratch by moving the whole forearm (high) – **10 secs**
225. Scratch with fingers only (high) – **10 secs**
226. Rubbing (high) – **10 secs**

c. Inner elbow (** Subject to lay non-dominant hand, palm up before starting)

231. Scratch by moving the whole forearm – **10 secs**
232. Scratch with fingers only – **10 secs**
233. Rubbing – **10 secs**
234. Scratch by moving the whole forearm (high) – **10 secs**
235. Scratch with fingers only (high) – **10 secs**
236. Rubbing (high) – **10 secs**

d. Palm

231. Scratch by moving the whole forearm – **10 secs**
232. Scratch with fingers only – **10 secs**
233. Rubbing – **10 secs**
234. Scratch by moving the whole forearm (high) – **10 secs**
235. Scratch with fingers only (high) – **10 secs**
236. Rubbing (high) – **10 secs**

e. Outer elbow (** Subject to bend elbow, with palm on the shoulder before starting)

251. Scratch by moving the whole forearm – **10 secs**
252. Scratch with fingers only – **10 secs**
253. Rubbing – **10 secs**
254. Scratch by moving the whole forearm (high) – **10 secs**
255. Scratch with fingers only (high) – **10 secs**
256. Rubbing (high) – **10 secs**

f. Shoulder

261. Scratch by moving the whole forearm – **10 secs**
262. Scratch with fingers only – **10 secs**
263. Rubbing – **10 secs**
264. Scratch by moving the whole forearm (high) – **10 secs**

265. Scratch with fingers only (high) – **10 secs**
266. Rubbing (high) – **10 secs**

g. Neck

271. Scratch by moving the whole forearm – **10 secs**
272. Scratch with fingers only – **10 secs**
273. Rubbing – **10 secs**
274. Scratch by moving the whole forearm (high) – **10 secs**
275. Scratch with fingers only (high) – **10** secs
276. Rubbing (high) – **10 secs**

h. Cheek

281. Scratch by moving the whole forearm – **10 secs**
282. Scratch with fingers only – **10 secs**
283. Rubbing – **10 secs**
284. Scratch by moving the whole forearm (high) – **10 secs**
285. Scratch with fingers only (high) – **10 secs**
286. Rubbing (high) – **10 secs**

i. Top of Head

291. Scratch by moving the whole forearm – **10 secs**
292. Scratch with fingers only – **10 secs**
293. Rubbing – **10 secs**
294. Scratch by moving the whole forearm (high) – **10 secs**
295. Scratch with fingers only (high) – **10 secs**
296. Rubbing (high) – **10 secs**

j. Calf

301. Scratch by moving the whole forearm – **10 secs**
302. Scratch with fingers only – **10 secs**
303. Rubbing – **10 secs**
304. Scratch by moving the whole forearm (high) – **10 secs**
305. Scratch with fingers only (high) – **10 secs**
306. Rubbing (high) – **10 secs**

k. Knee

311. Scratch by moving the whole forearm – **10 secs**
312. Scratch with fingers only – **10 secs**
313. Rubbing – **10 secs**
314. Scratch by moving the whole forearm (high) – **10 secs**
315. Scratch with fingers only (high) – **10 secs**
316. Rubbing (high) – **10 secs**

l. Top of thigh

321. Scratch by moving the whole forearm – **10 secs**
322. Scratch with fingers only – **10 secs**
323. Rubbing – **10 secs**
324. Scratch by moving the whole forearm (high) – **10 secs**
325. Scratch with fingers only (high) – **10 secs**
326. Rubbing (high) – **10 secs**

m. Abdomen

331. Scratch by moving the whole forearm – **10 secs**
332. Scratch with fingers only – **10 secs**
333. Rubbing – **10 secs**
334. Scratch by moving the whole forearm (high) – **10 secs**
335. Scratch with fingers only (high) – **10 secs**
336. Rubbing (high) – **10 secs**

Part 3 Scratching intensities

*** User is to start at resting position before each activity, and to return to resting position after each activity.*

***Higher intensity = Harder and faster*

1. Scratching with articulating arm – See recommended set
2. Scratching with fingers only – See recommended set

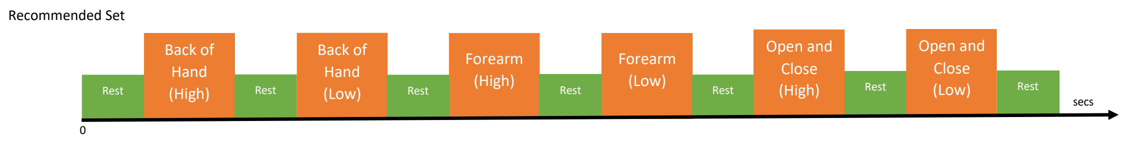

Sample data

Part 0 Resting position

011 (Rest forearm on table with elbow hanging at 90 degrees, palm facing down and resting on the surface of the table – 10 secs)

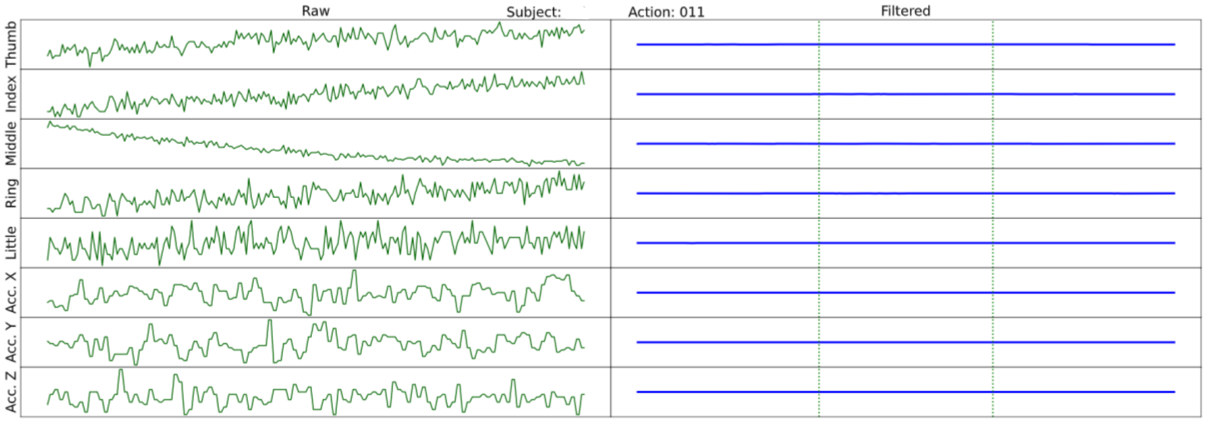

Figure 1. 011 (Rest forearm on table with elbow hanging at 90 degrees, palm facing down and resting on the surface of the table – 10 secs)

Part 0 Closed grip

021 (Curl all the fingers as much as possible except the thumb, relax thumb, “Thumbs up” – 5 secs)

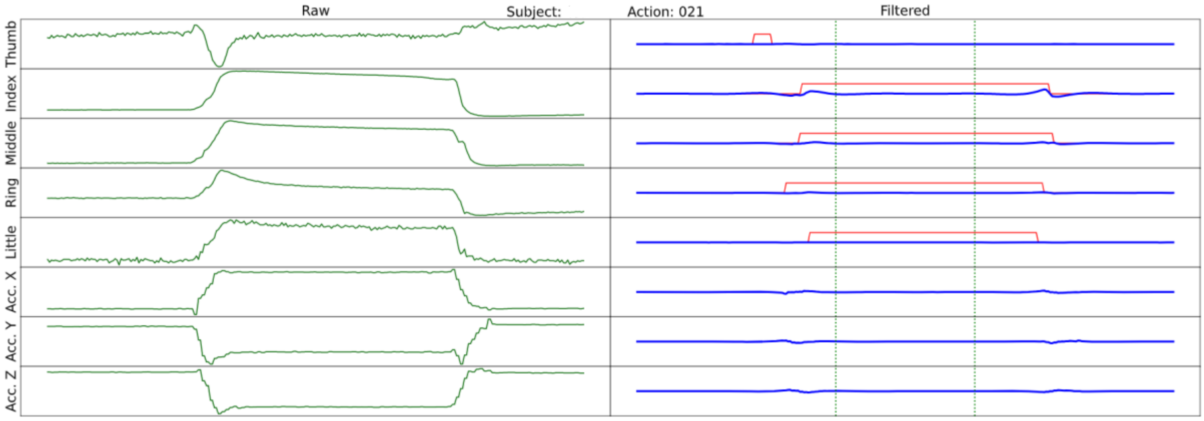

Figure 2. 021 (Curl all the fingers as much as possible except the thumb, relax thumb, “Thumbs up” – 5 secs)

022 (Curl thumb as much as possible and relax the other fingers, “Show 4” – 5 secs)

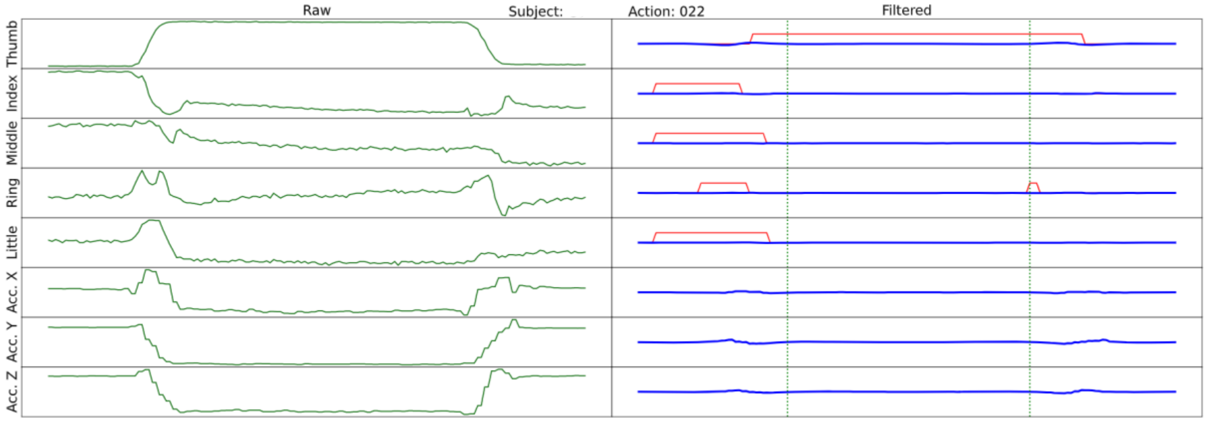

Figure 3. 022 (Curl thumb as much as possible and relax the other fingers, “Show 4” – 5 secs)

Part 1 Non-scratching activities:

111 (Forearm hanging, waving the arm side-to-side, palm facing down – 10 secs)

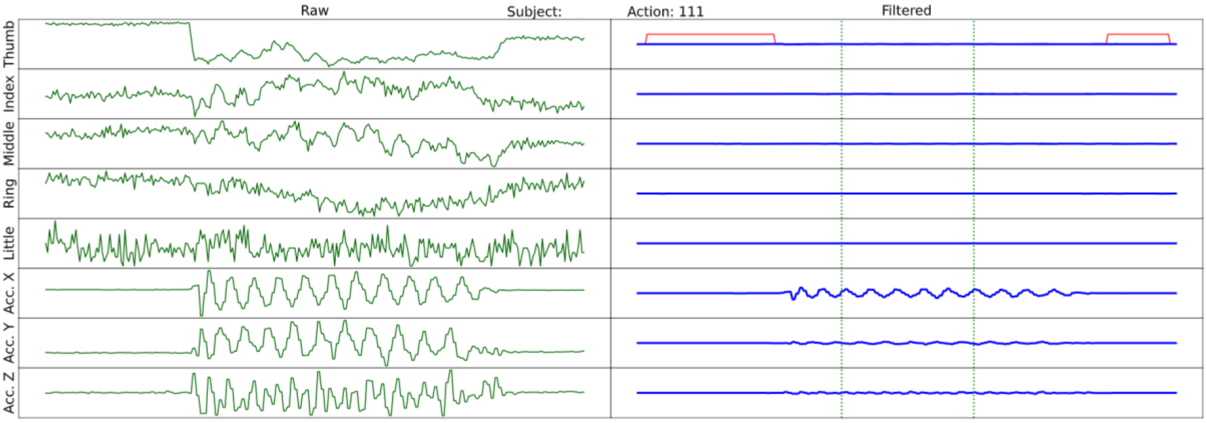

Figure 4. 111 (Forearm hanging, waving the arm side-to-side, palm facing down – 10 secs)

112 (Forearm hanging, waving the arm side-to-side, palm facing medially – 10 secs)

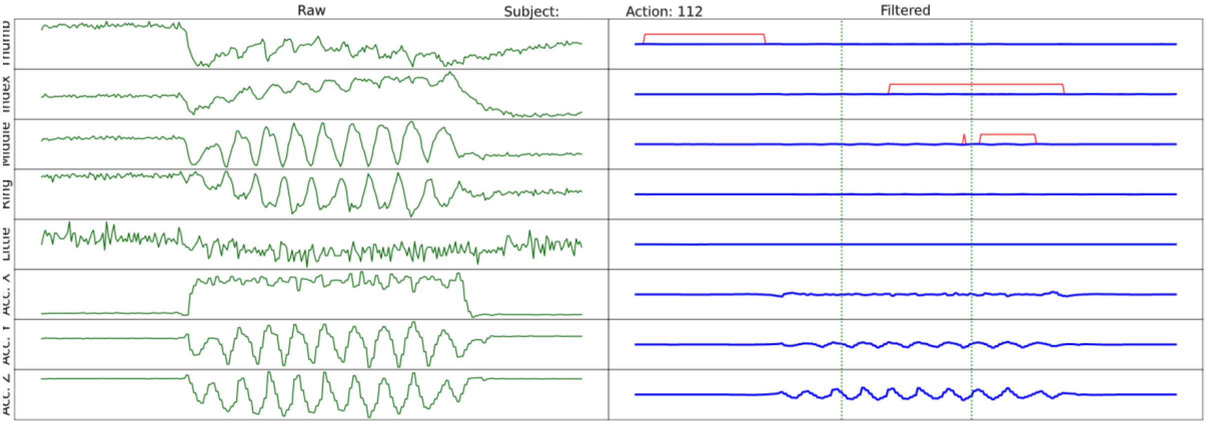

Figure 5. 112 (Forearm hanging, waving the arm side-to-side, palm facing medially – 10 secs)

113 (Forearm hanging, waving the arm side-to-side, palm facing forward – 10 secs)

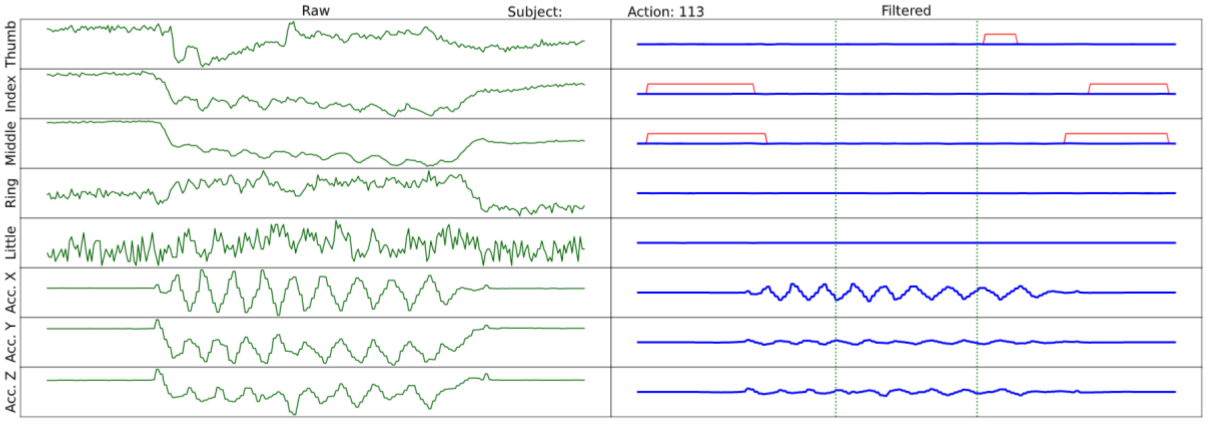

Figure 6. 113 (Forearm hanging, waving the arm side-to-side, palm facing forward – 10 secs)

114 (Forearm hanging, waving the arm forward-and-back, palm facing forward – 10 secs)

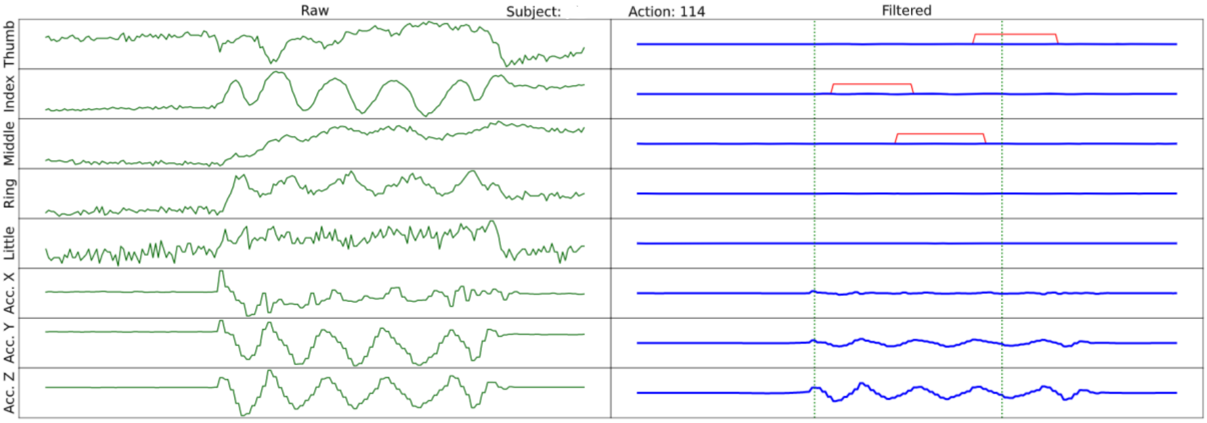

Figure 7. 114 (Forearm hanging, waving the arm forward-and-back, palm facing forward – 10 secs)

115 (Forearm rested, waving palm facing down, side-to-side – 10 secs)

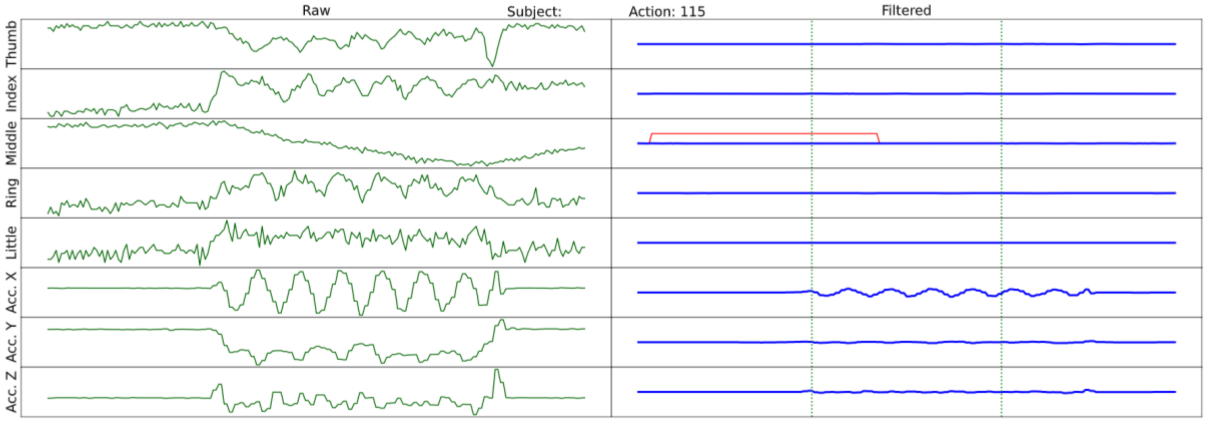

Figure 8. 115 (Forearm rested, waving palm facing down, side-to-side – 10 secs)

116 (Forearm rested, waving palm facing medially, side-to-side – 10 secs)

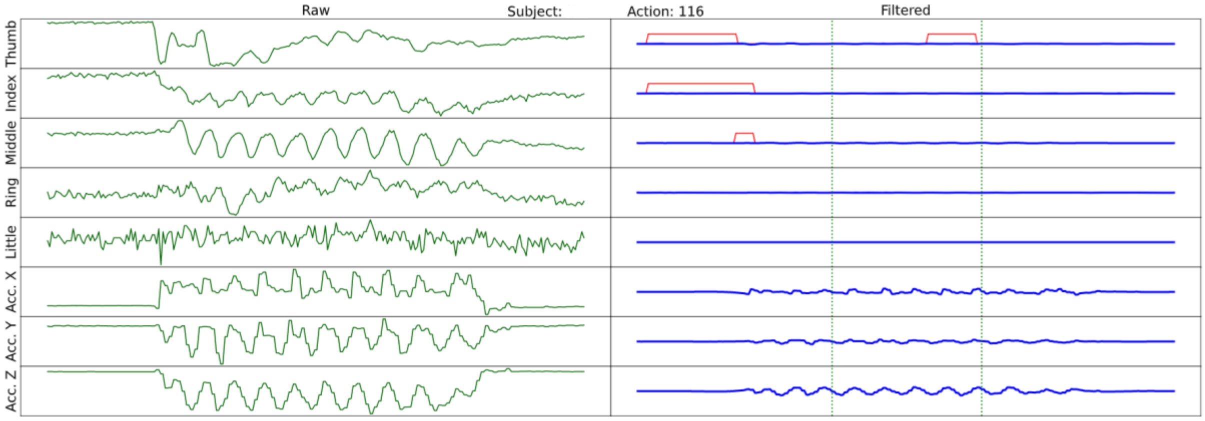

Figure 9. 116 (Forearm rested, waving palm facing medially, side-to-side – 10 secs)

117 (Forearm rested, waving palm facing forward, side-to-side – 10 secs)

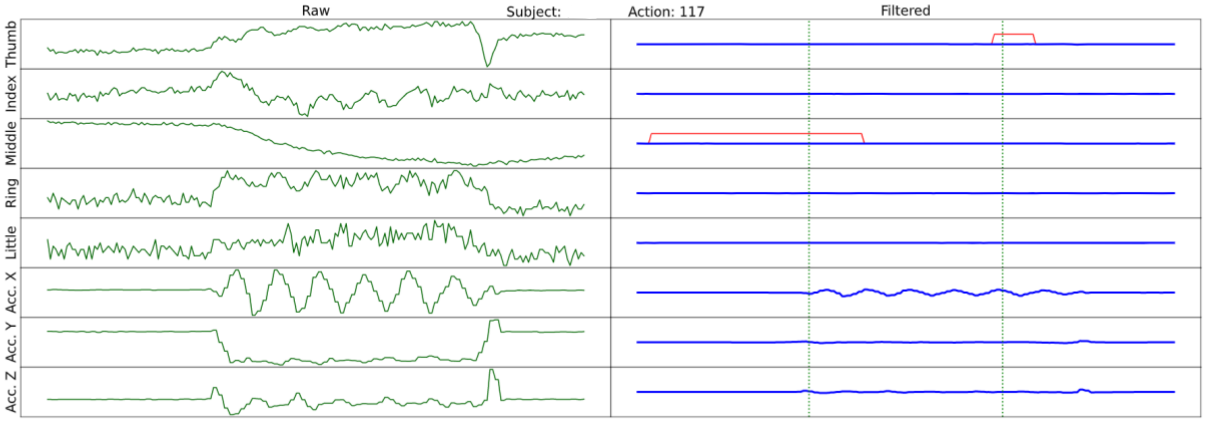

Figure 10. 117 (Forearm rested, waving palm facing forward, side-to-side – 10 secs)

118 (Forearm rested, waving palm facing forward, forward-and-back – 10 secs)

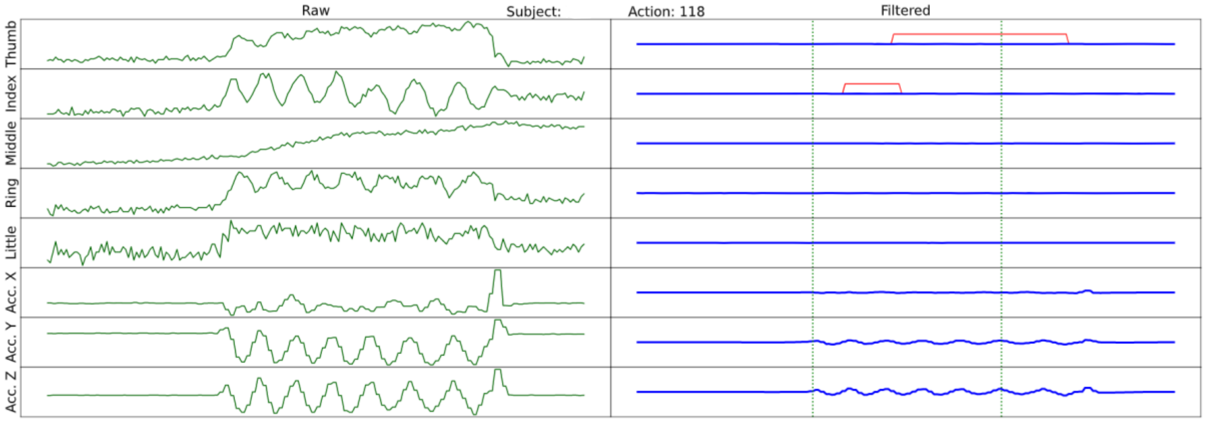

Figure 11. 118 (Forearm rested, waving palm facing forward, forward-and-back – 10 secs)

121 (Typing a message on a smartphone – until completion)

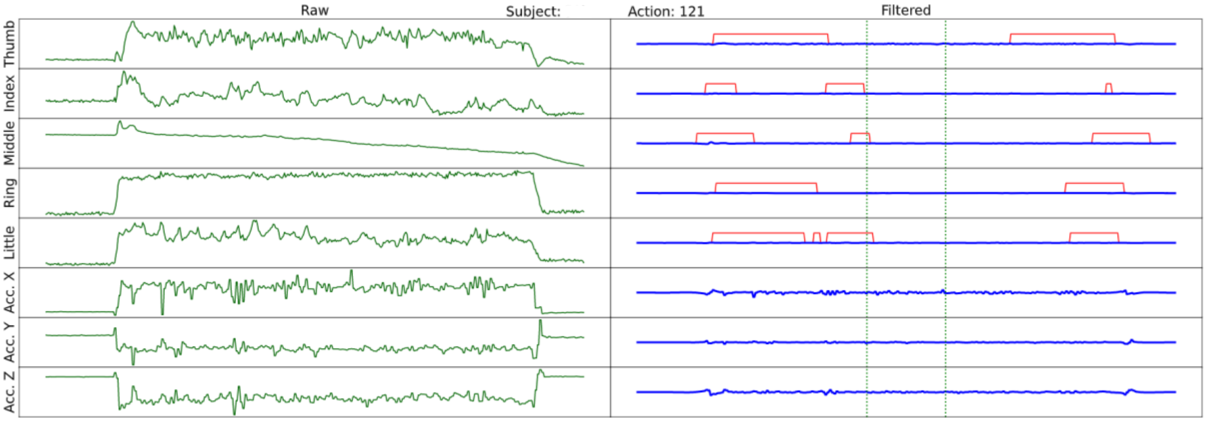

Figure 12. 121 (Typing a message on a smartphone – until completion)

122 (Typing a message on a keyboard – until completion)

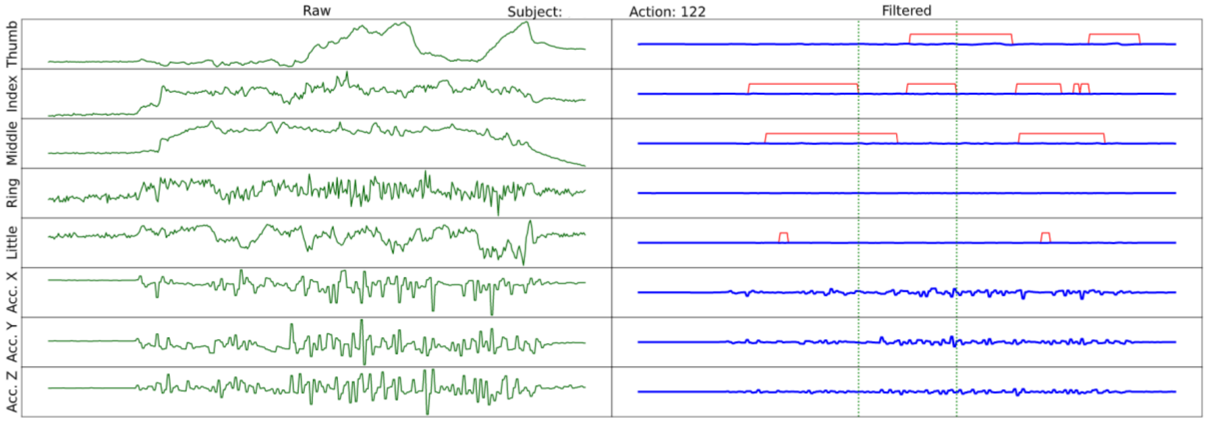

Figure 13. 122 (Typing a message on a keyboard – until completion)

131 (Tapping with palm resting on table, fingers extended – 10 secs)

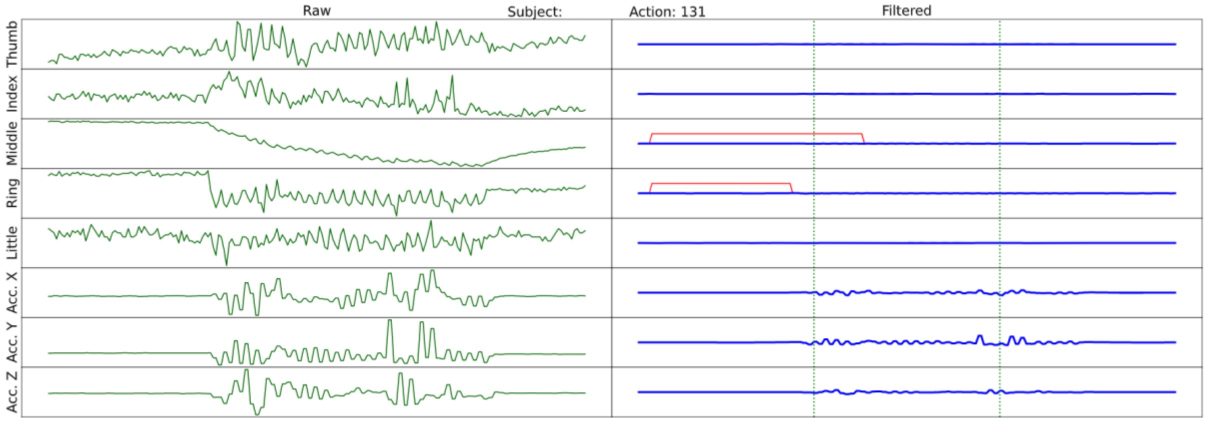

Figure 14. 131 (Tapping with palm resting on table, fingers extended – 10 secs)

132 (Tapping with palm resting on table, fingers bent – 10 secs)

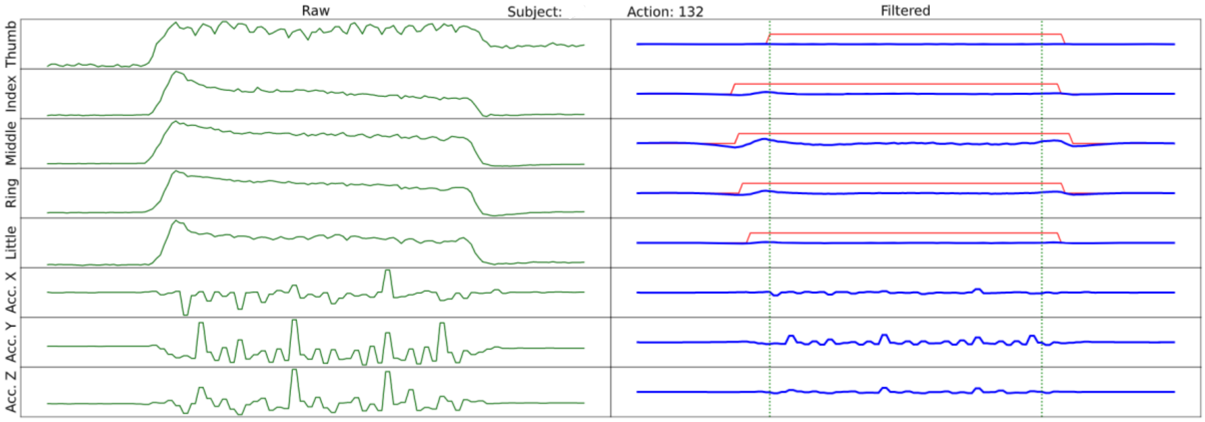

Figure 15. 132 (Tapping with palm resting on table, fingers bent – 10 secs)

141 (Grabbing a can, and shaking it up-and-down – 10 secs)

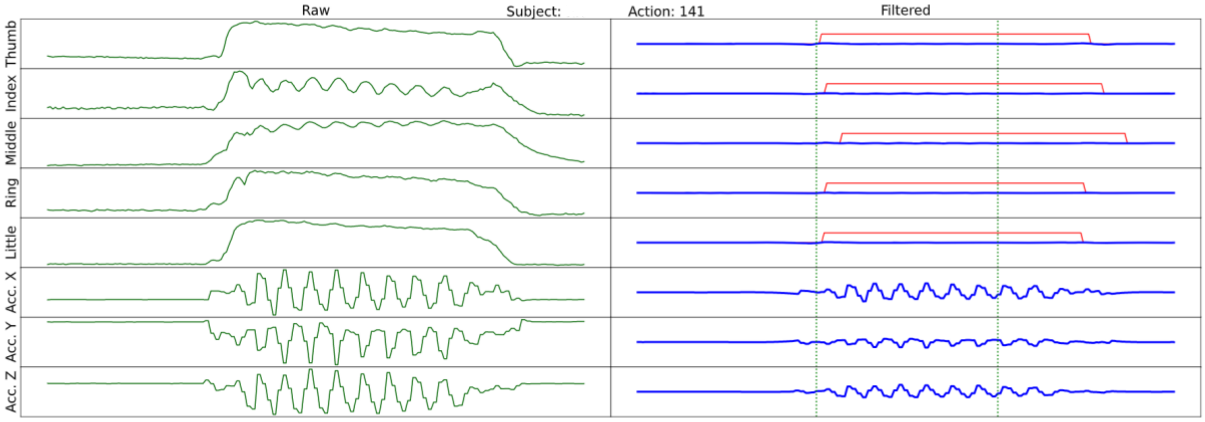

Figure 16. 141 (Grabbing a can, and shaking it up-and-down – 10 secs)

142 (Grabbing a can, and shaking it side-to-side – 10 secs)

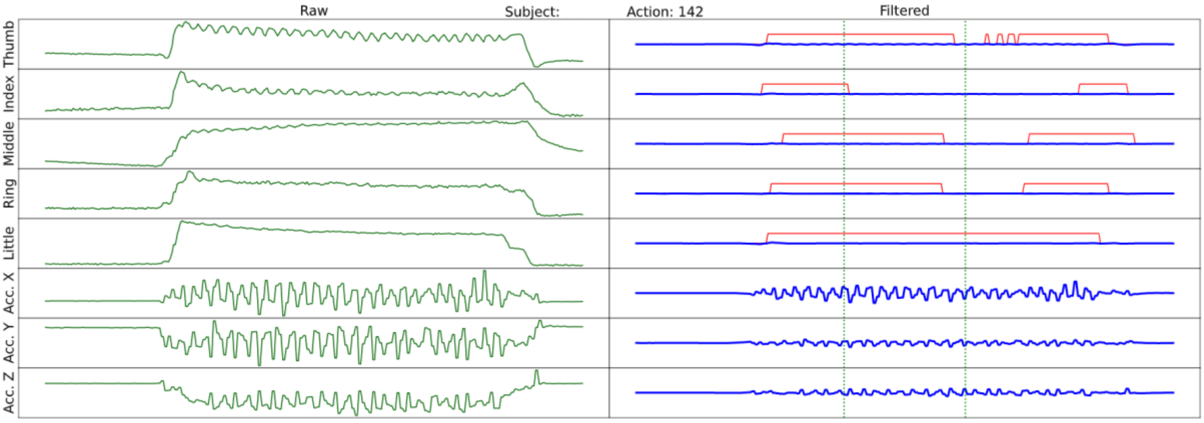

Figure 17. 142 (Grabbing a can, and shaking it side-to-side – 10 secs)

151 (Grabbing a can and transfer it over a partition – 10 secs)

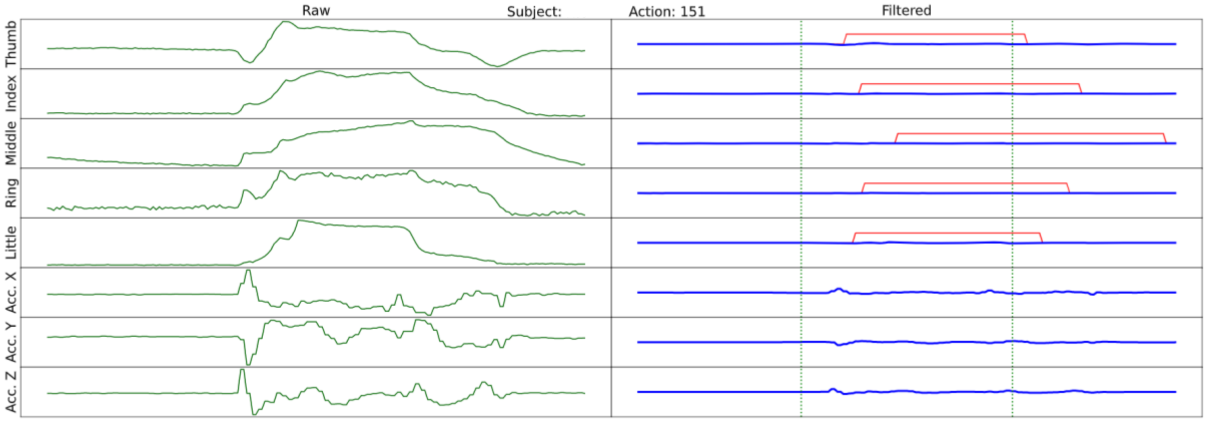

Figure 18. 151 (Grabbing a can and transfer it over a partition – 10 secs)

152 (Grabbing a can and transfer it over a partition – 10 secs)

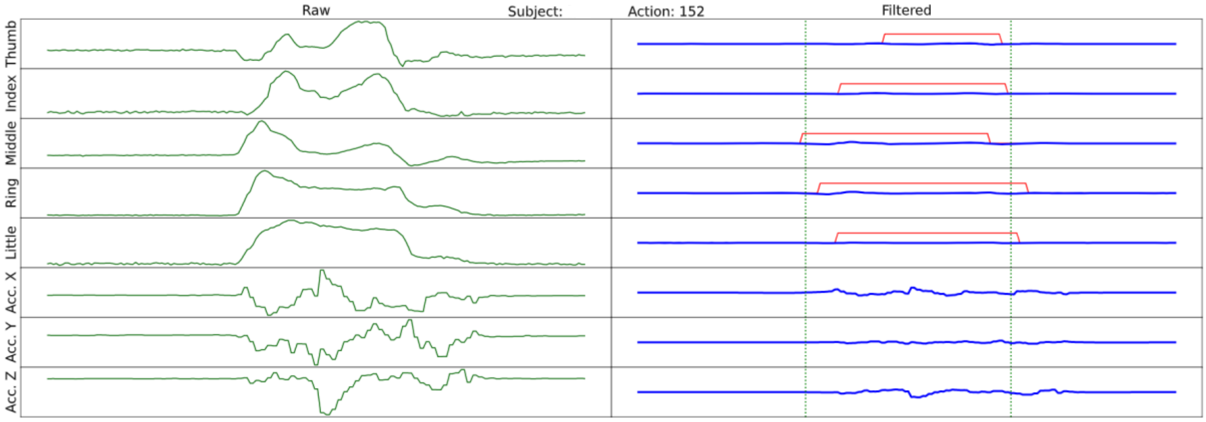

Figure 19. 152 (Grabbing a can and transfer it over a partition – 10 secs)

161 (Turning the key to open a lock – until completion)

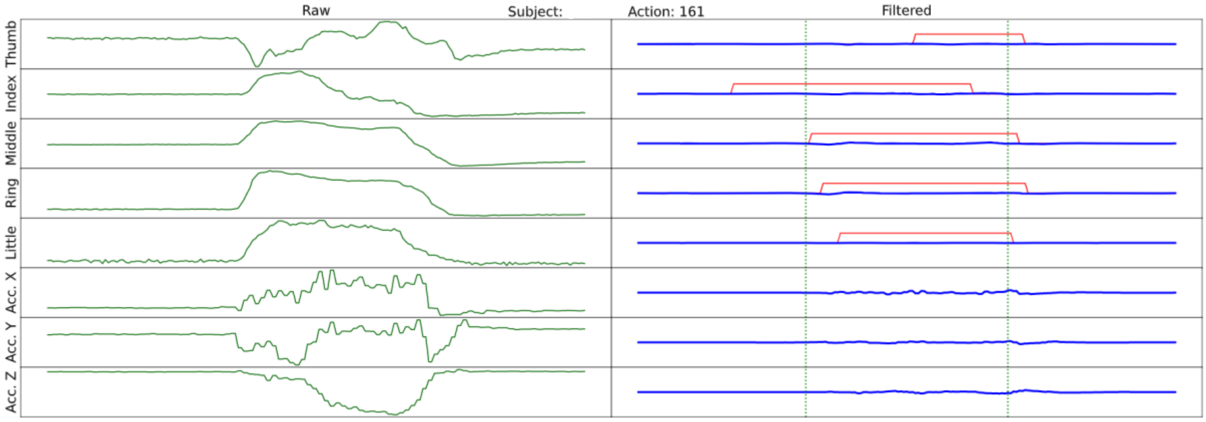

Figure 20. 161 (Turning the key to open a lock – until completion)

171 (Separate a bottle from its cap, and place both onto table – until completion)

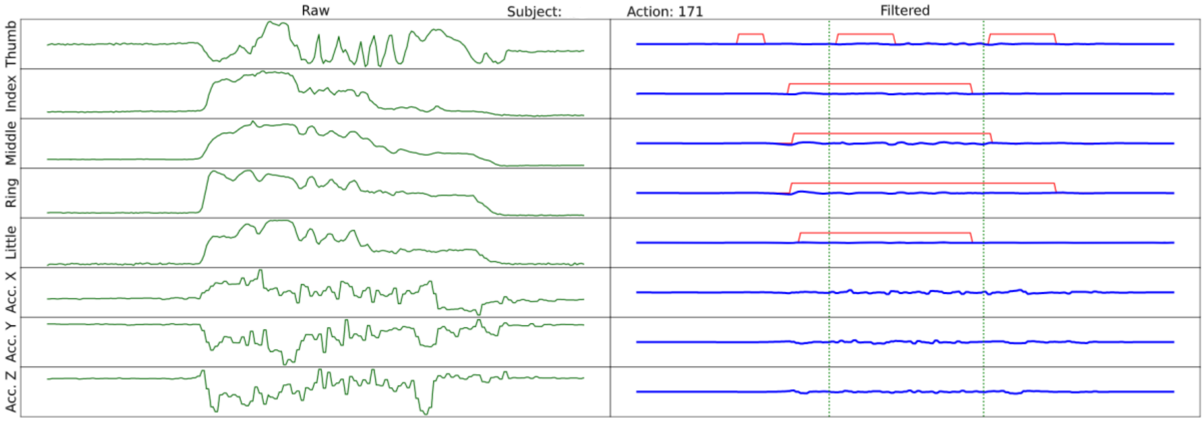

Figure 21. 171 (Separate a bottle from its cap, and place both onto table – until completion)

172 (Fit cap back onto the bottle, and place both onto table – until completion)

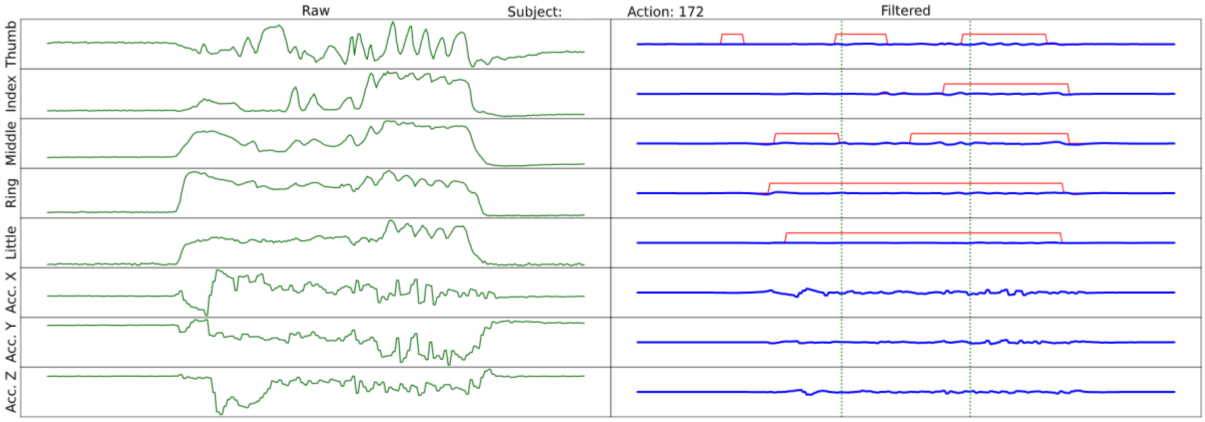

Figure 22. 172 (Fit cap back onto the bottle, and place both onto table – until completion)

Part 2 Scratching activities

211 (Scratch back of hand by only moving the whole forearm – 10 secs)

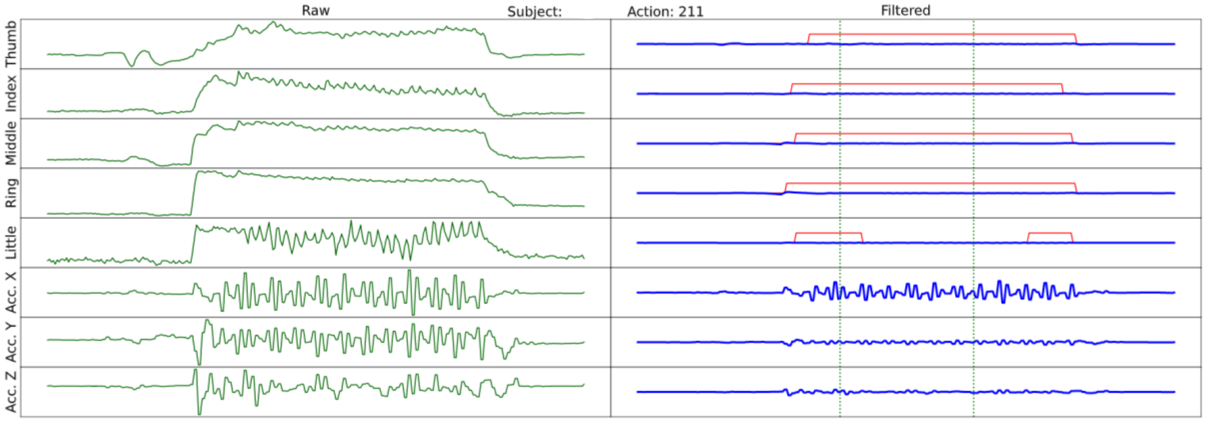

Figure 23. 211 (Scratch back of hand by moving the whole forearm – 10 sec)

212 (Scratch back of hand by only moving the fingers – 10 secs)

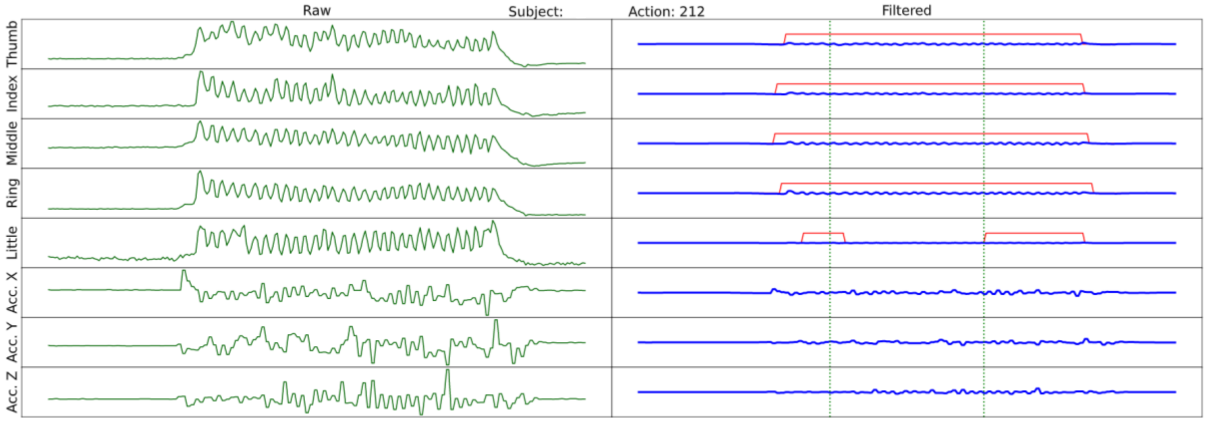

Figure 24. 212 (Scratch back of hand by only moving the fingers – 10 secs)

213 (Rubbing back of hand – 10 secs)

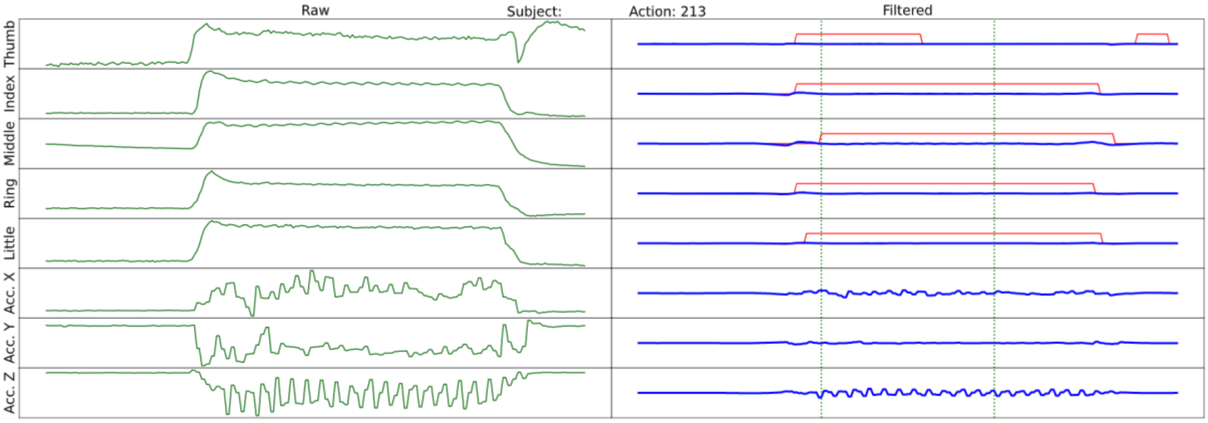

Figure 25. 213 (Rubbing back of hand – 10 secs)

214 (Scratch back of hand by only moving the whole forearm with high intensity – 10 secs)

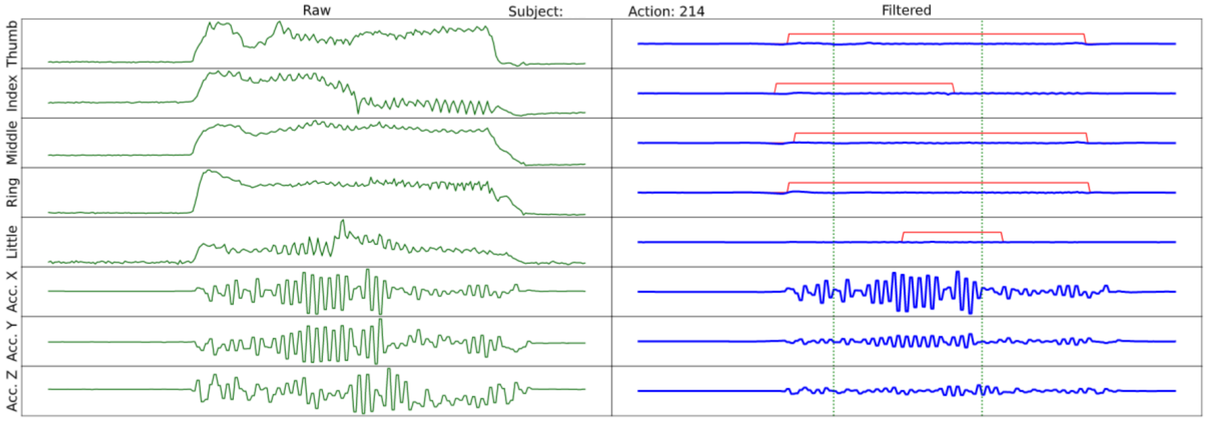

Figure 26. 214 (Scratch back of hand by only moving the whole forearm with high intensity – 10 secs)

215 (Scratch back of hand by only moving the fingers with high intensity – 10 secs)

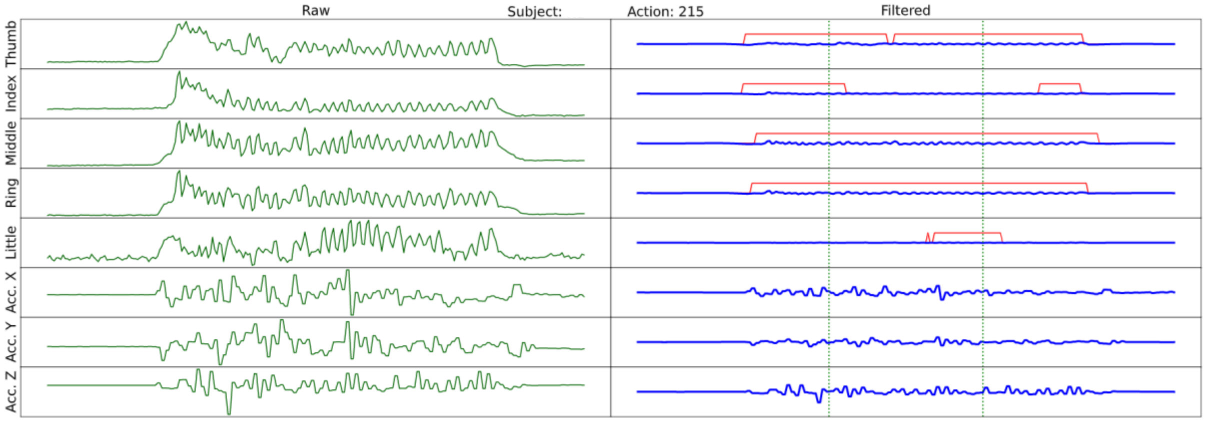

Figure 27. 215 (Scratch back of hand by only moving the fingers with high intensity – 10 secs)

216 (Rubbing back of hand with high intensity – 10 secs)

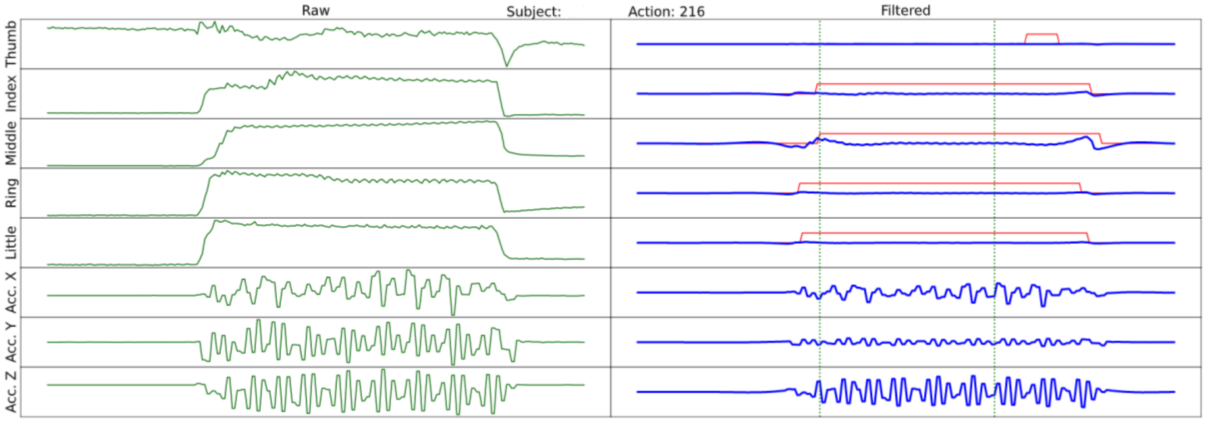

Figure 28. 216 (Rubbing back of hand with high intensity – 10 secs)

221 (Scratch forearm by only moving the whole forearm – 10 secs)

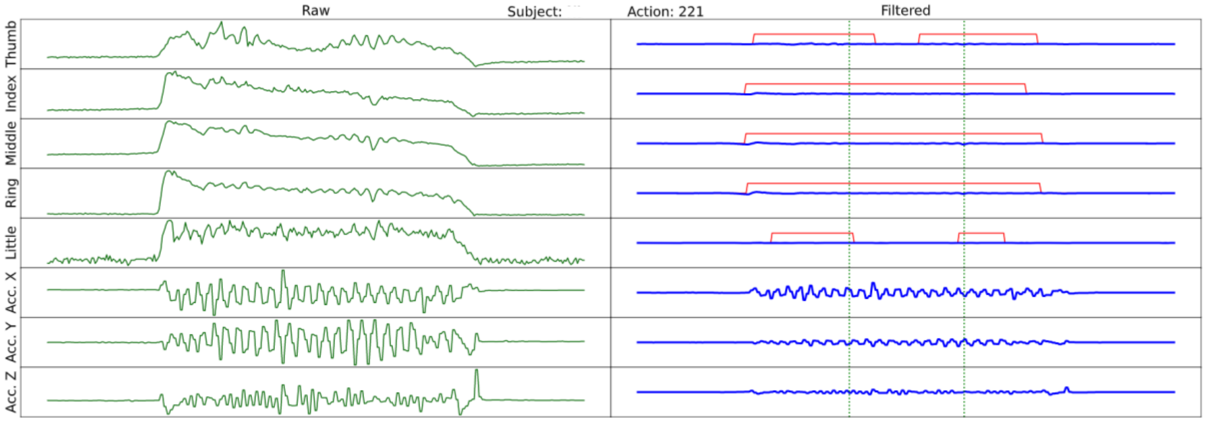

Figure 29. 221 (Scratch forearm by only moving the whole forearm – 10 secs)

222 (Scratch forearm by only moving the fingers – 10 secs)

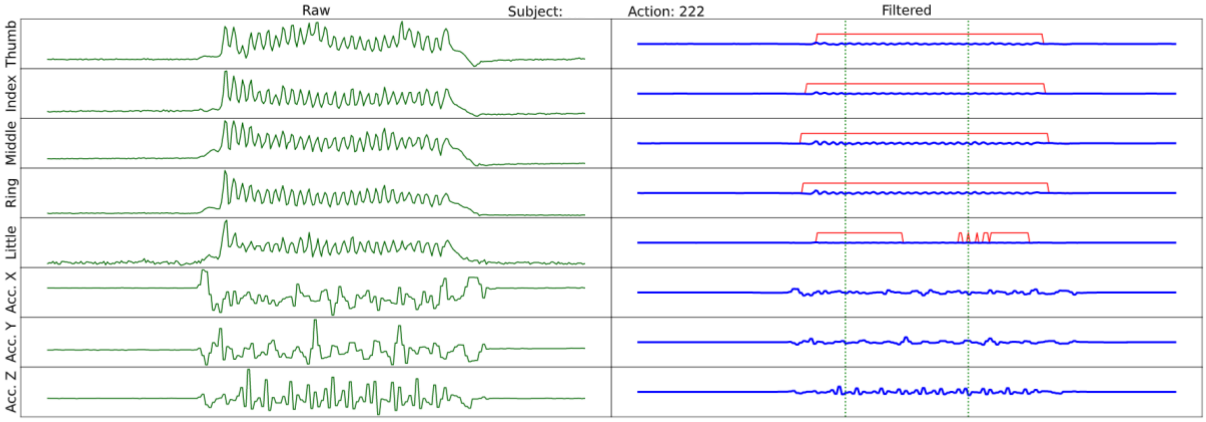

Figure 30. 222 (Scratch forearm by only moving the fingers – 10 secs)

223 (Rubbing forearm – 10 secs)

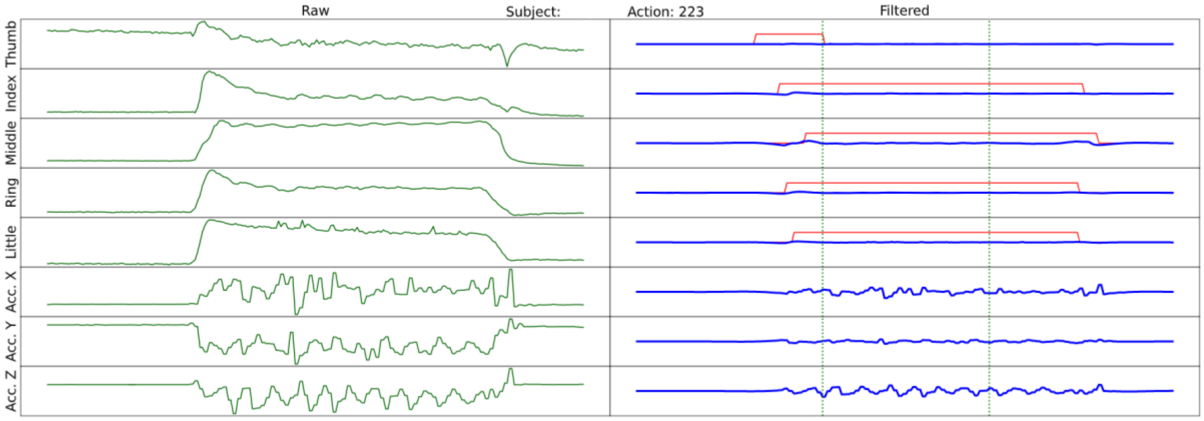

Figure 31. 223 (Rubbing forearm – 10 secs)

224 (Scratch forearm by only moving the whole forearm with high intensity – 10 secs)

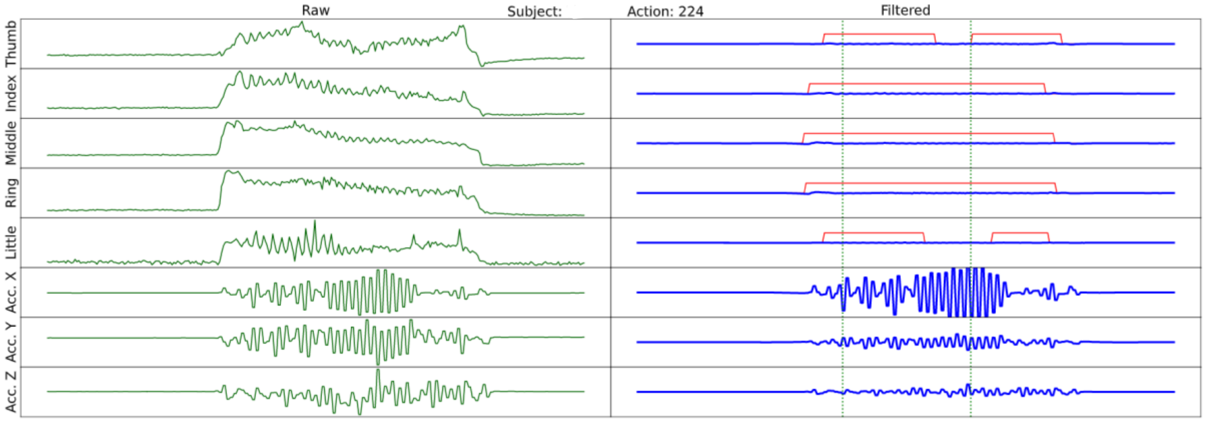

Figure 32. 224 (Scratch forearm by only moving the whole forearm with high intensity – 10 secs)

225 (Scratch forearm by only moving the fingers with high intensity – 10 secs)

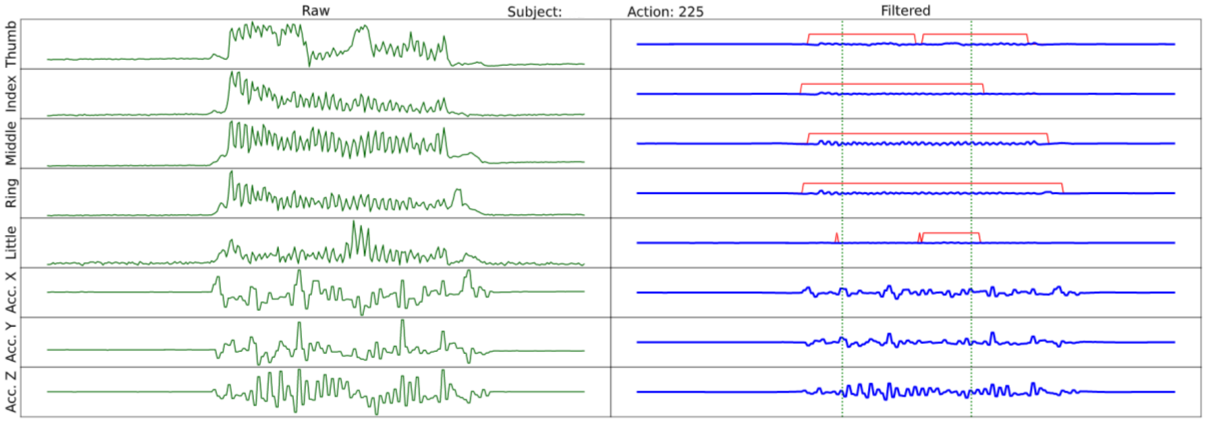

Figure 33. 225 (Scratch forearm by only moving the fingers with high intensity – 10 secs)

226 (Rubbing forearm with high intensity – 10 secs)

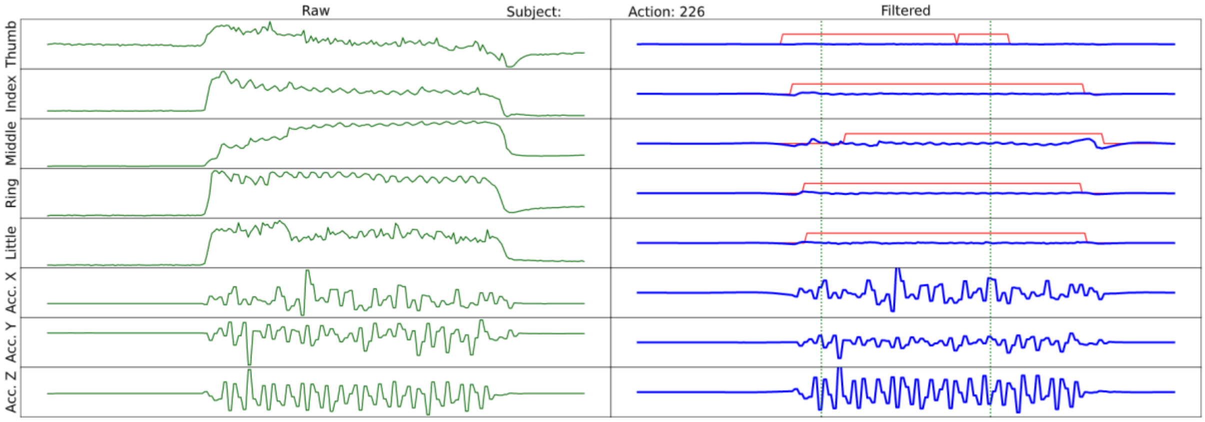

Figure 34. 226 (Rubbing forearm with high intensity – 10 secs)

231 (Scratch inner elbow by only moving the whole forearm – 10 secs)

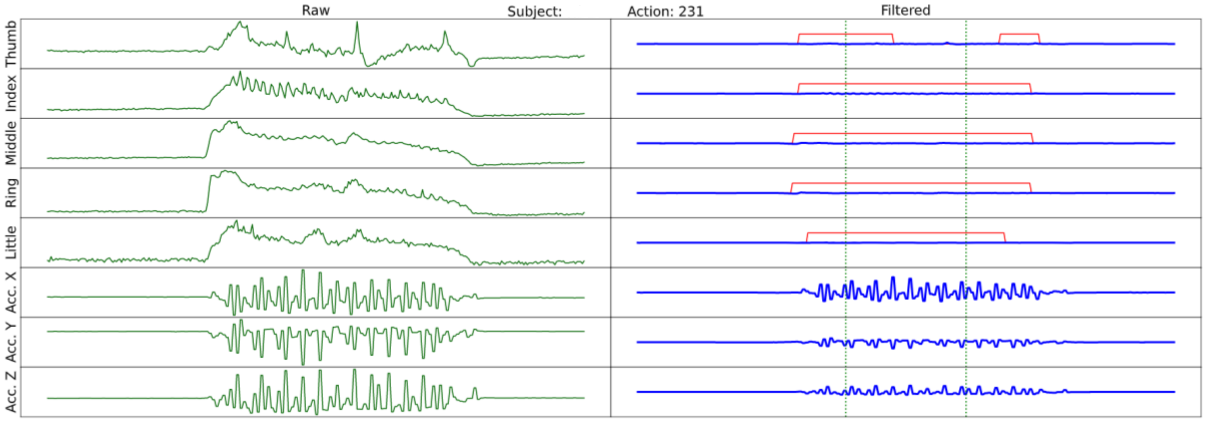

Figure 35. 231 (Scratch inner elbow by only moving the whole forearm – 10 secs)

232 (Scratch inner elbow by only moving the fingers – 10 secs)

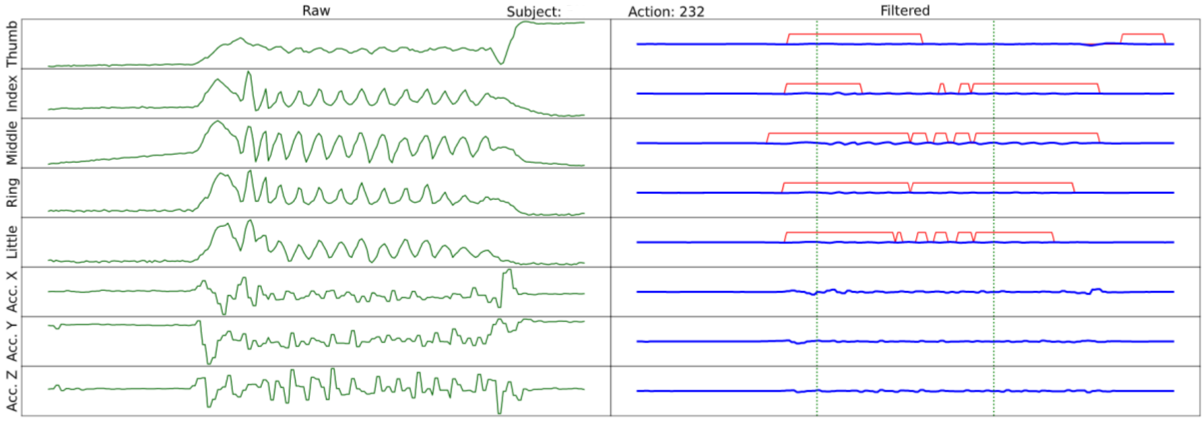

Figure 36. 232 (Scratch inner elbow by only moving the fingers – 10 secs)

233 (Rubbing inner elbow – 10 secs)

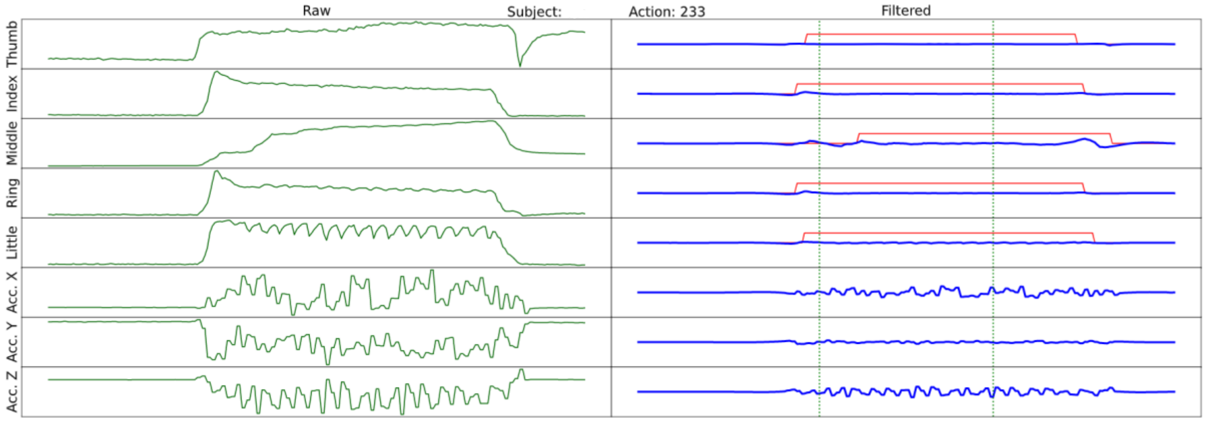

Figure 37. 233 (Rubbing inner elbow – 10 secs)

234 (Scratch inner elbow by only moving the whole forearm with high intensity – 10 secs)

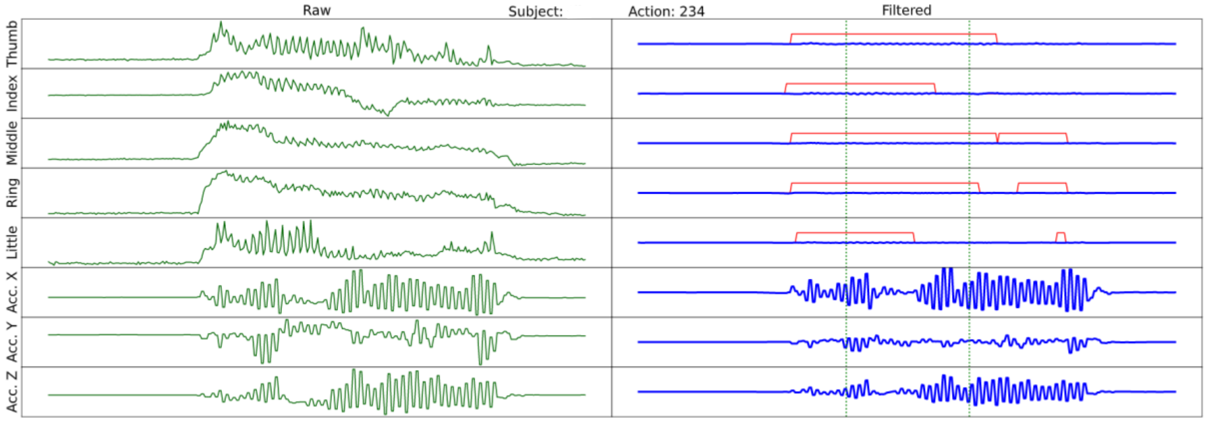

Figure 38. 234 (Scratch inner elbow by only moving the whole forearm with high intensity – 10 secs)

235 (Scratch inner elbow by only moving the fingers with high intensity – 10 secs)

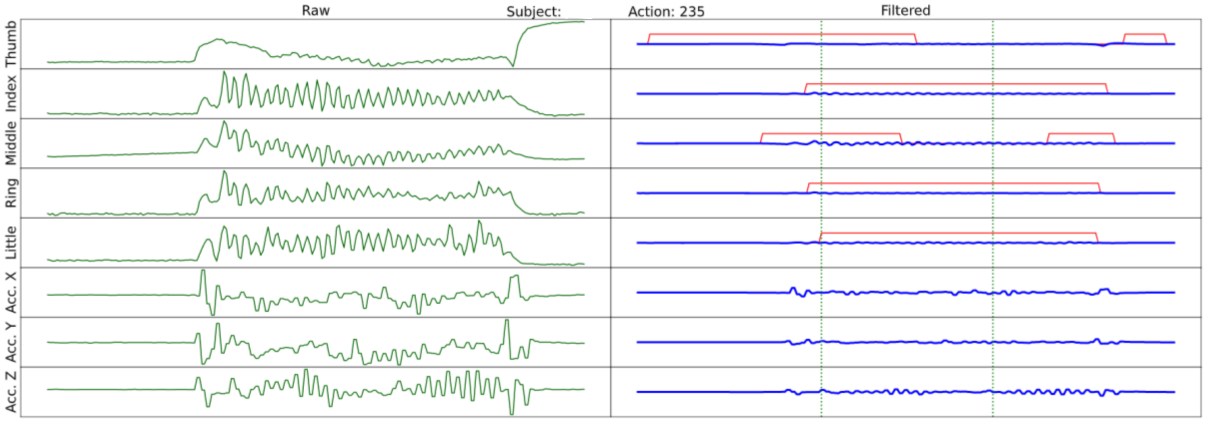

Figure 39. 235 (Scratch inner elbow by only moving the fingers with high intensity – 10 secs)

236 (Rubbing inner elbow with high intensity – 10 secs)

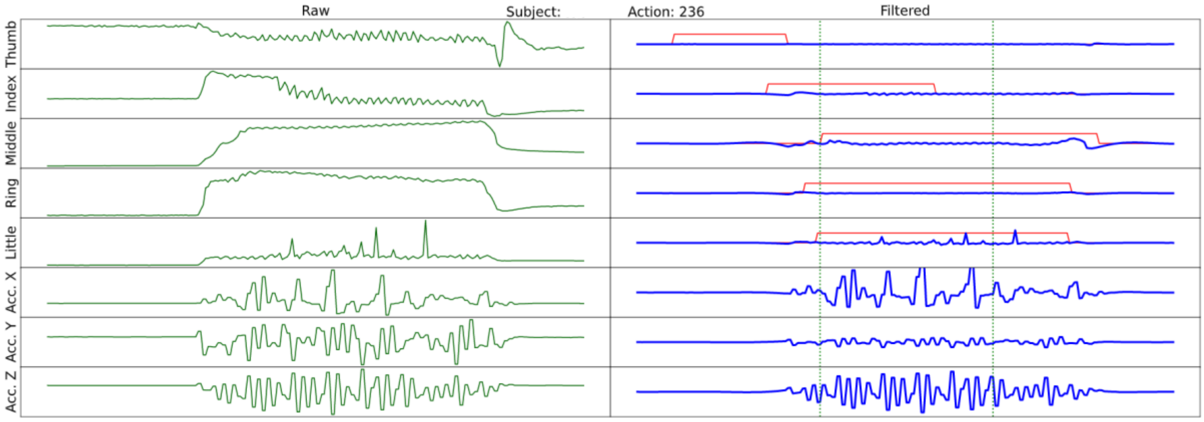

Figure 40. 236 (Rubbing inner elbow with high intensity – 10 secs)

241 (Scratch palm by only moving the whole forearm – 10 secs)

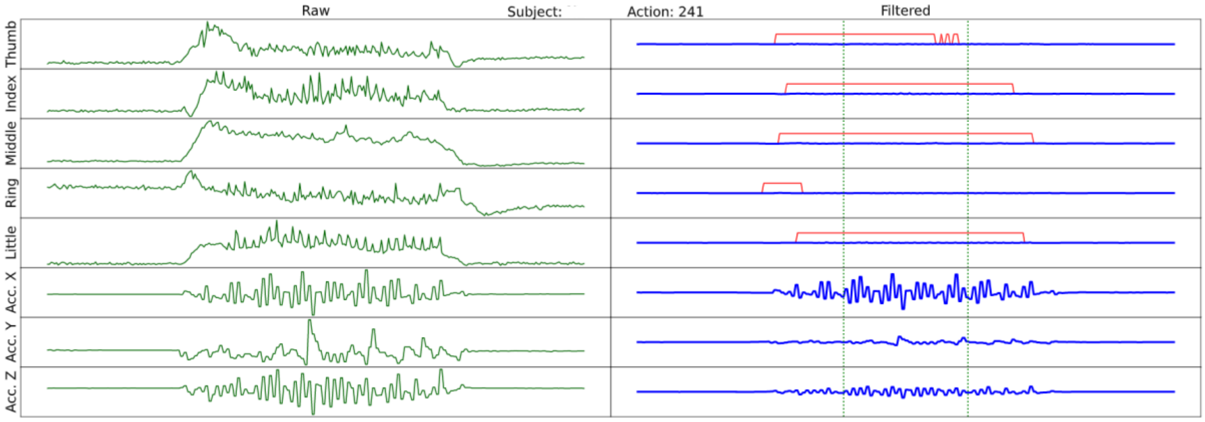

Figure 41. 241 (Scratch palm by only moving the whole forearm – 10 secs)

242 (Scratch palm by only moving the fingers – 10 secs)

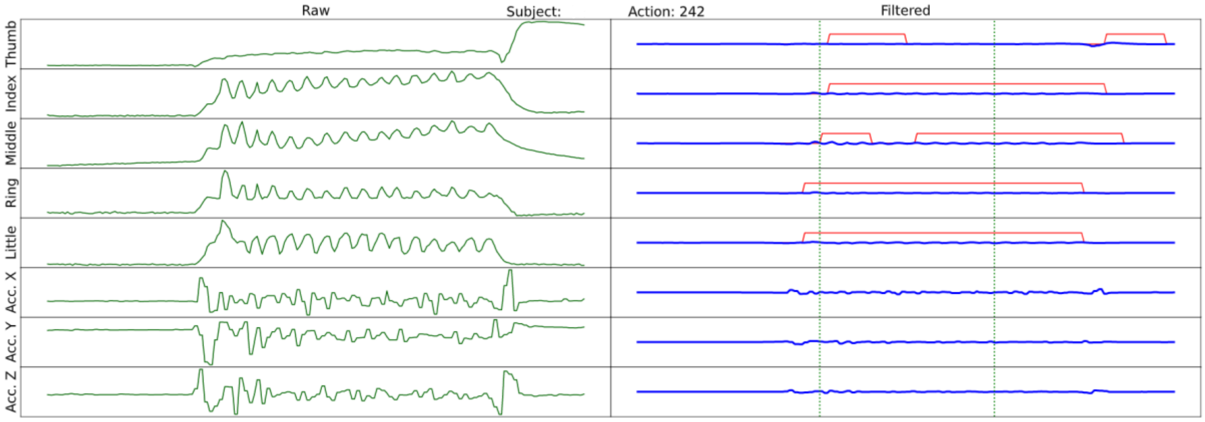

Figure 42. 242 (Scratch palm by only moving the fingers – 10 secs)

243 (Rubbing palm – 10 secs)

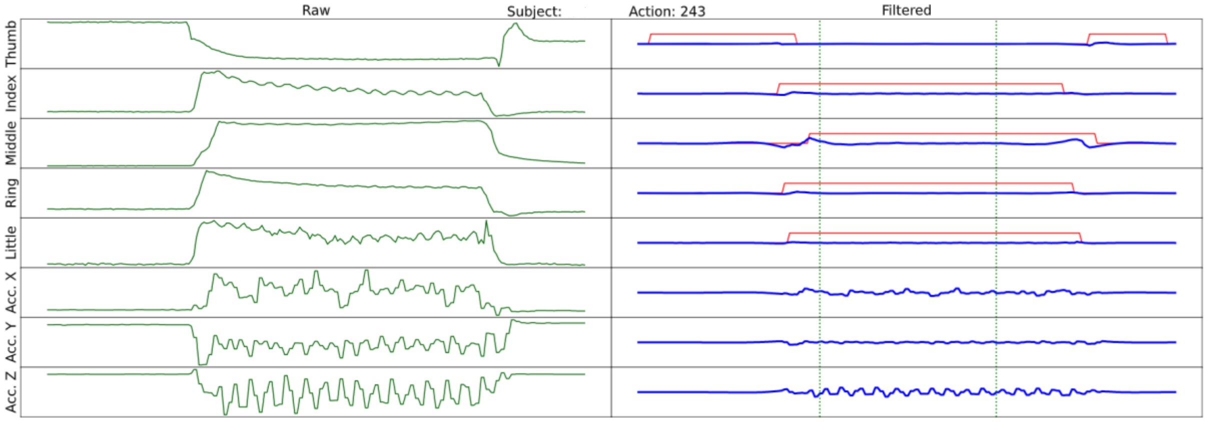

Figure 43. 243 (Rubbing palm – 10 secs)

244 (Scratch palm by only moving the whole forearm with high intensity – 10 secs)

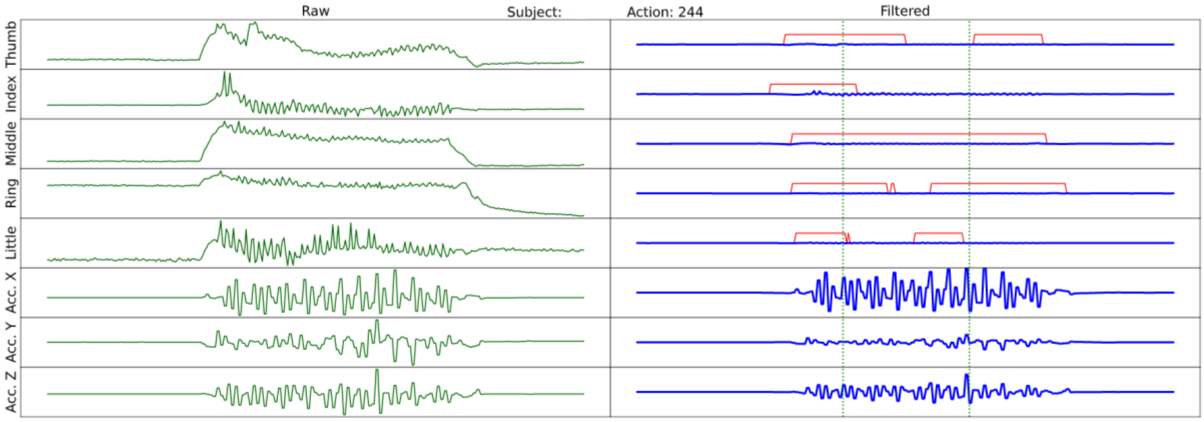

Figure 44. 244 (Scratch palm by only moving the whole forearm with high intensity – 10 secs)

245 (Scratch palm by only moving the fingers with high intensity – 10 secs)

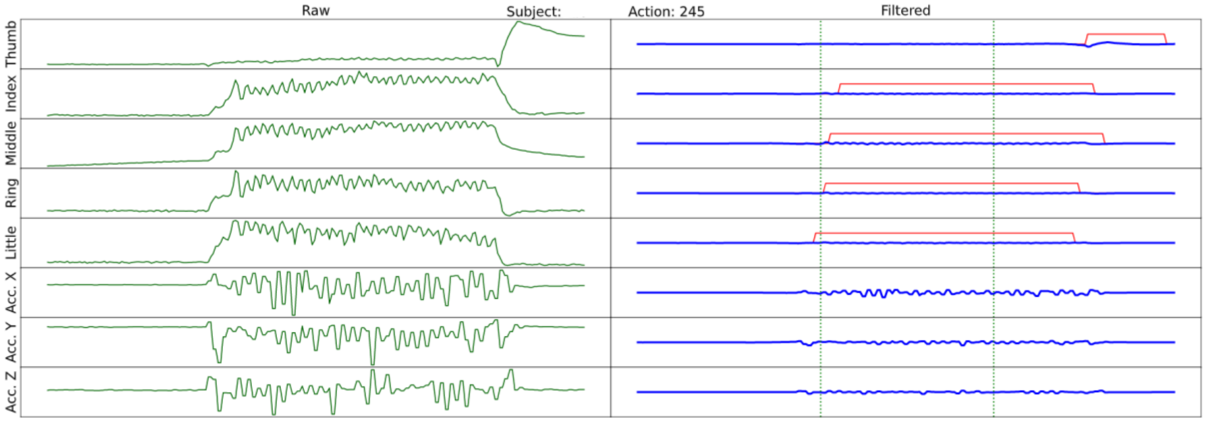

Figure 45. 245 (Scratch palm by only moving the fingers with high intensity – 10 secs)

246 (Rubbing palm with high intensity – 10 secs)

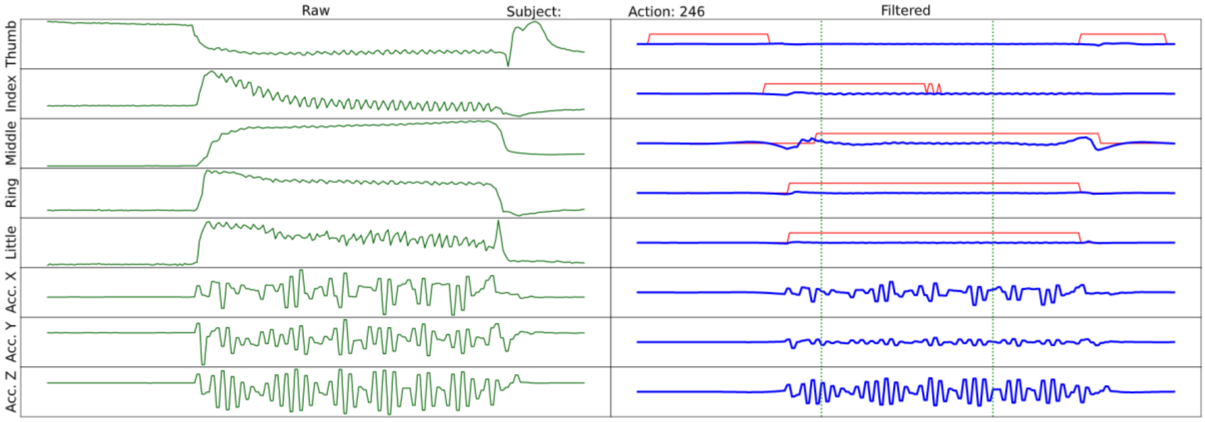

Figure 46. 246 (Rubbing palm with high intensity – 10 secs)

251 (Scratch outer elbow by only moving the whole forearm – 10 secs)

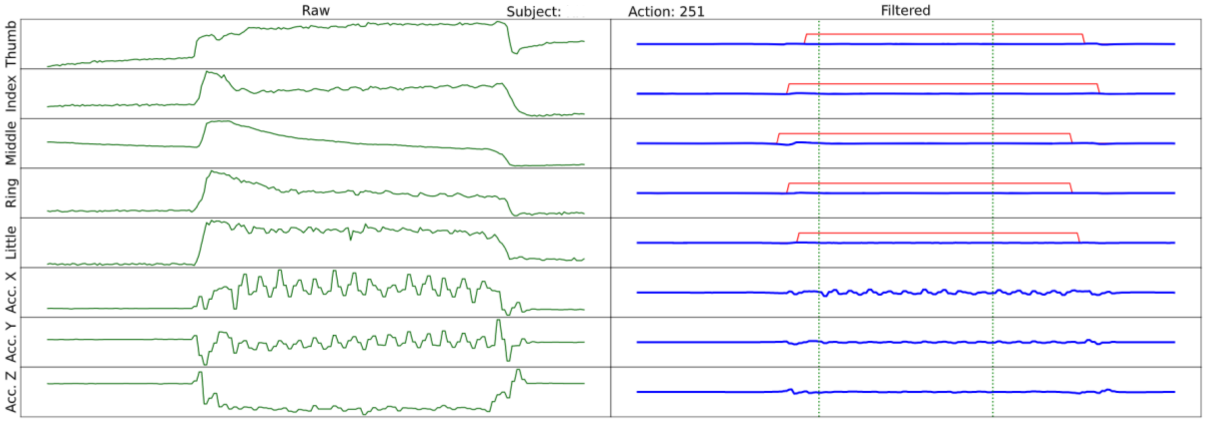

Figure 47. 251 (Scratch outer elbow by only moving the whole forearm – 10 secs)

252 (Scratch outer elbow by only moving the fingers – 10 secs)

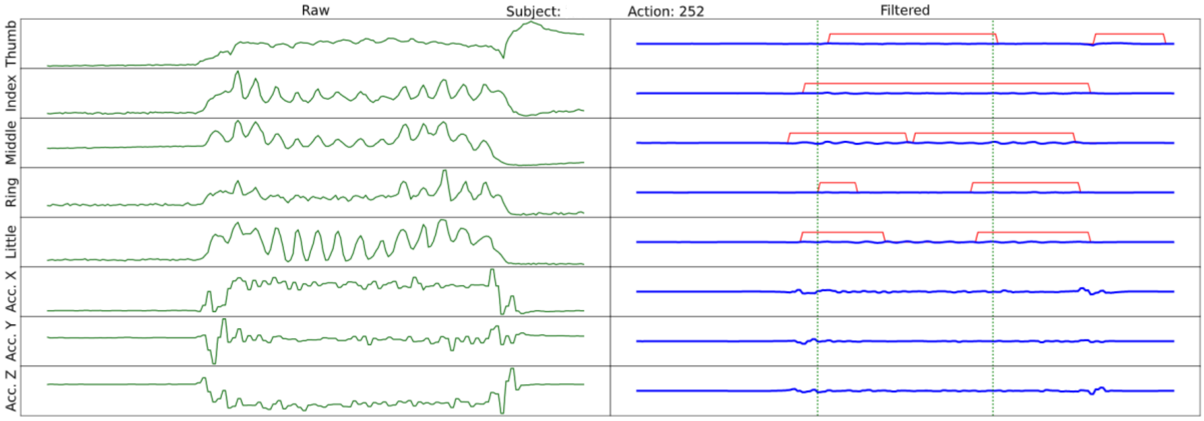

Figure 48. 252 (Scratch outer elbow by only moving the fingers – 10 secs)

253 (Rubbing outer elbow – 10 secs)

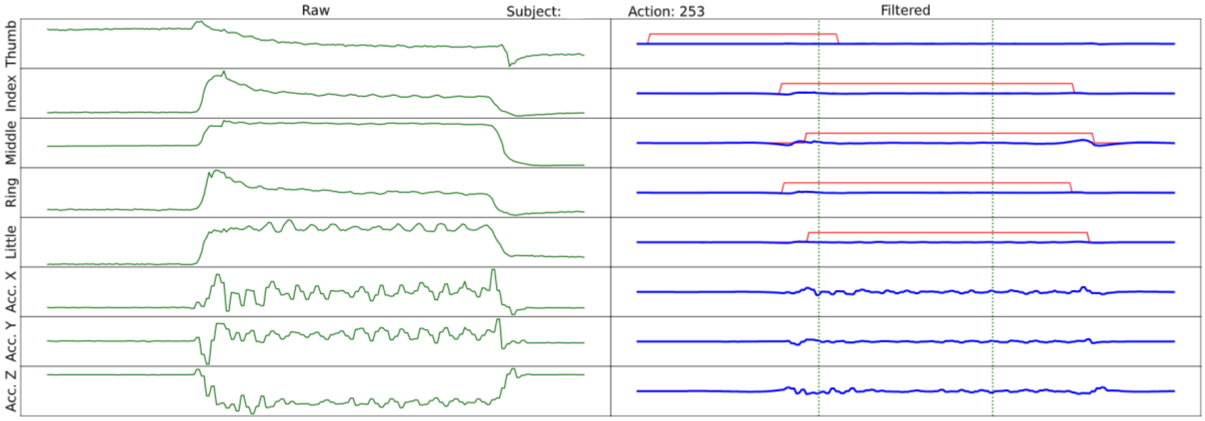

Figure 49. 253 (Rubbing outer elbow – 10 secs)

254 (Scratch outer elbow by only moving the whole forearm with high intensity – 10 secs)

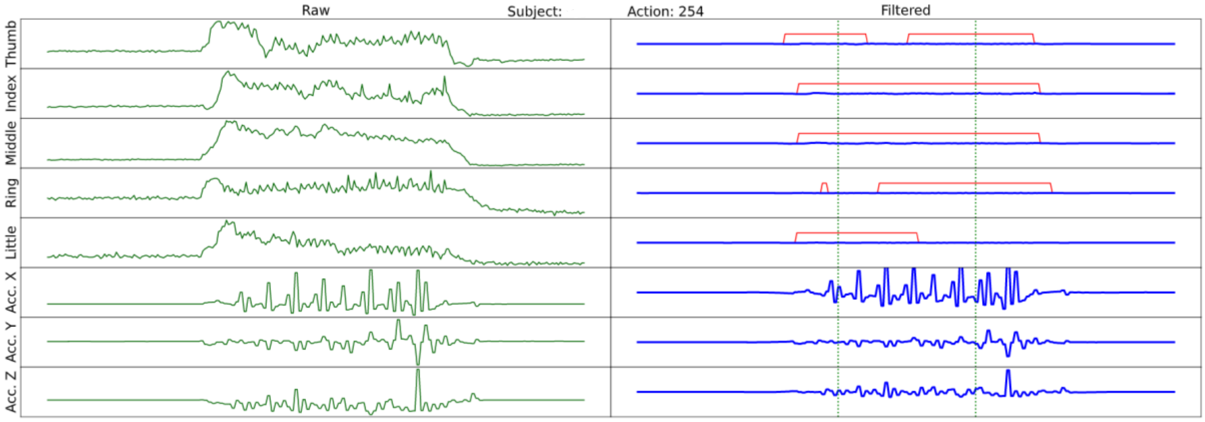

Figure 50. 254 (Scratch outer elbow by only moving the whole forearm with high intensity – 10 secs)

255 (Scratch outer elbow by only moving the fingers with high intensity – 10 secs)

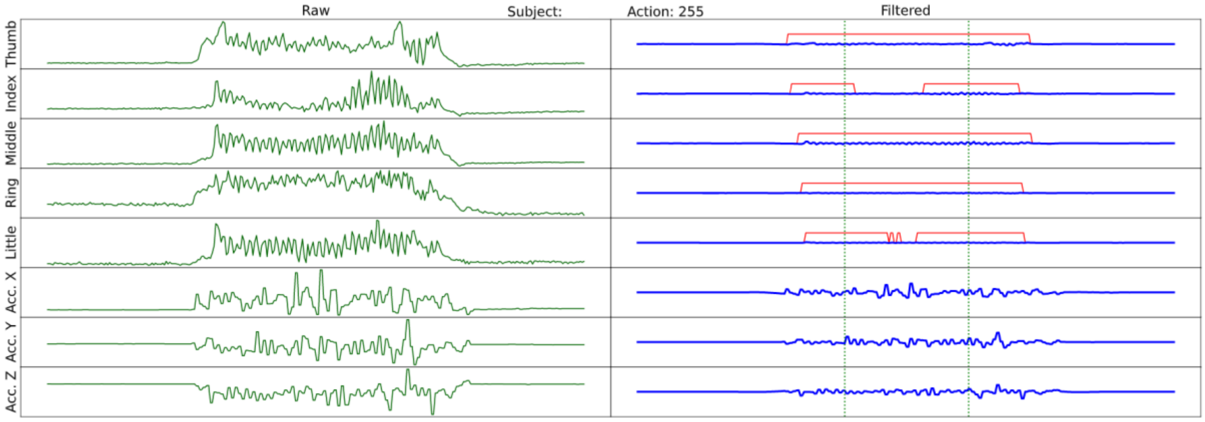

Figure 51. 255 (Scratch outer elbow by only moving the fingers with high intensity – 10 secs)

256 (Rubbing outer elbow with high intensity – 10 secs)

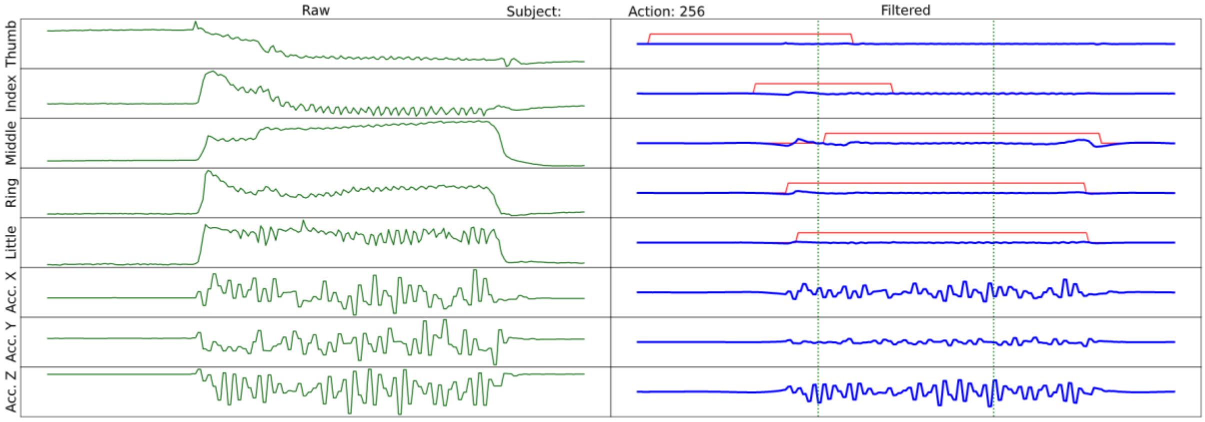

Figure 52. 256 (Rubbing outer elbow with high intensity – 10 secs)

261 (Scratch shoulder by only moving the whole forearm – 10 secs)

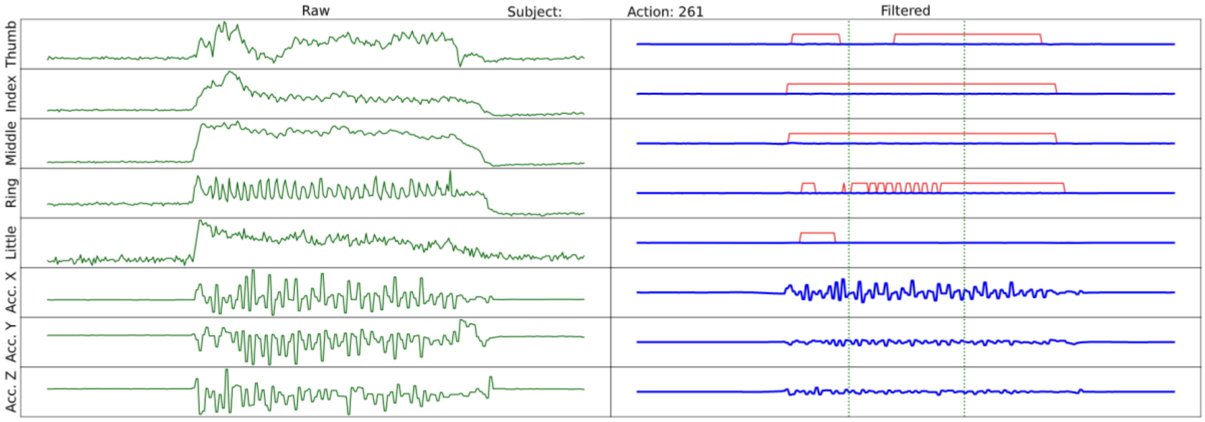

Figure 53. 261 (Scratch shoulder by only moving the whole forearm – 10 secs)

262 (Scratch shoulder by only moving the fingers – 10 secs)

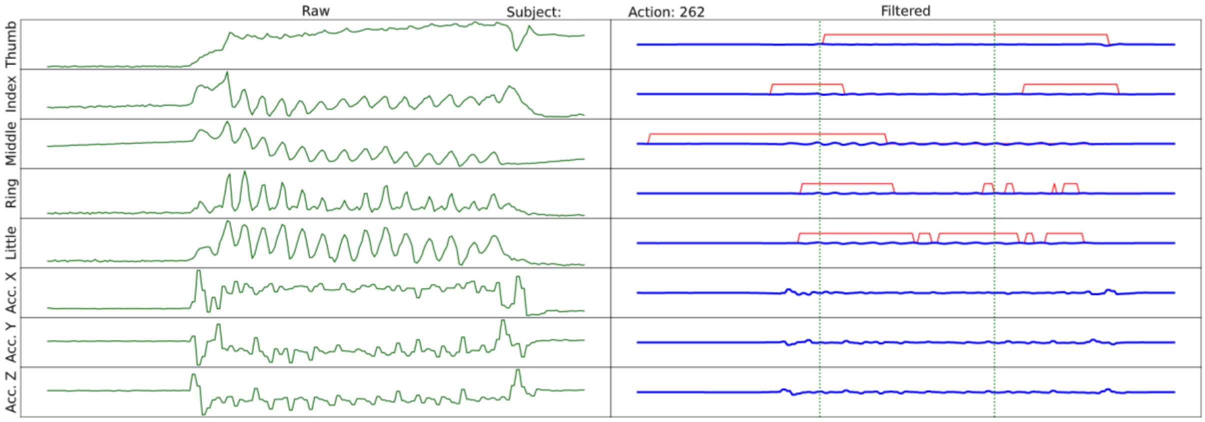

Figure 54. 262 (Scratch shoulder by only moving the fingers – 10 secs)

263 (Rubbing shoulder – 10 secs)

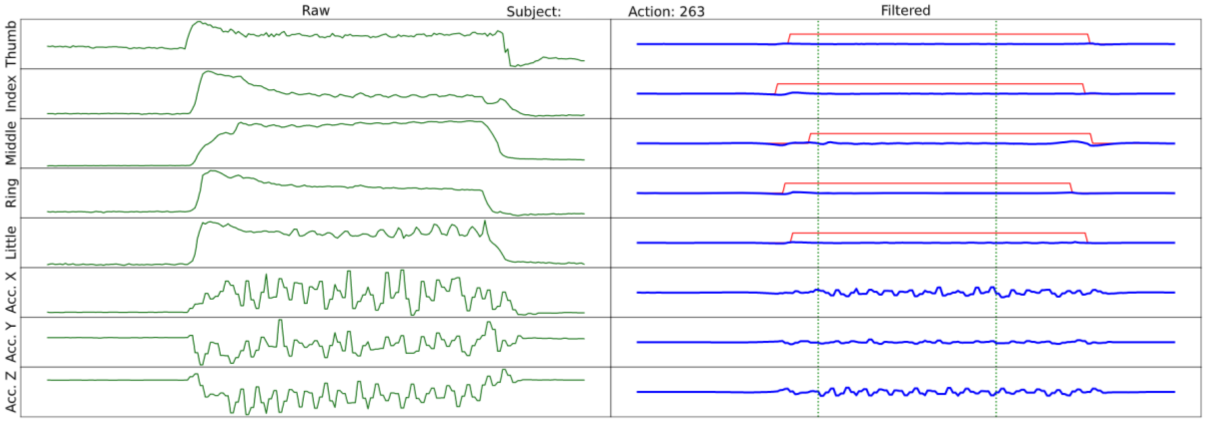

Figure 55. 263 (Rubbing shoulder – 10 secs)

264 (Scratch shoulder by only moving the whole forearm with high intensity – 10 secs)

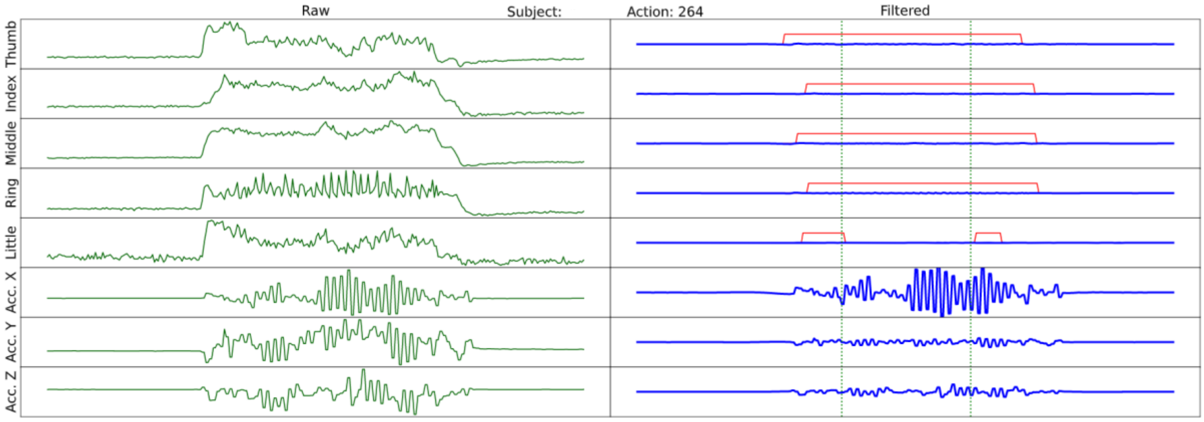

Figure 56. 264 (Scratch shoulder by only moving the whole forearm with high intensity – 10 secs)

265 (Scratch shoulder by only moving the fingers with high intensity – 10 secs)

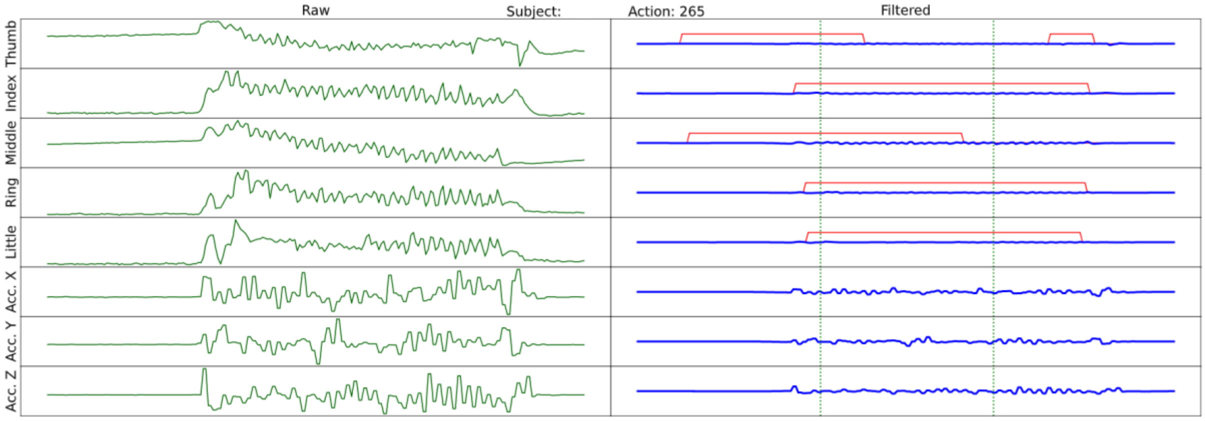

Figure 57. 265 (Scratch shoulder by only moving the fingers with high intensity – 10 secs)

266 (Rubbing shoulder with high intensity – 10 secs)

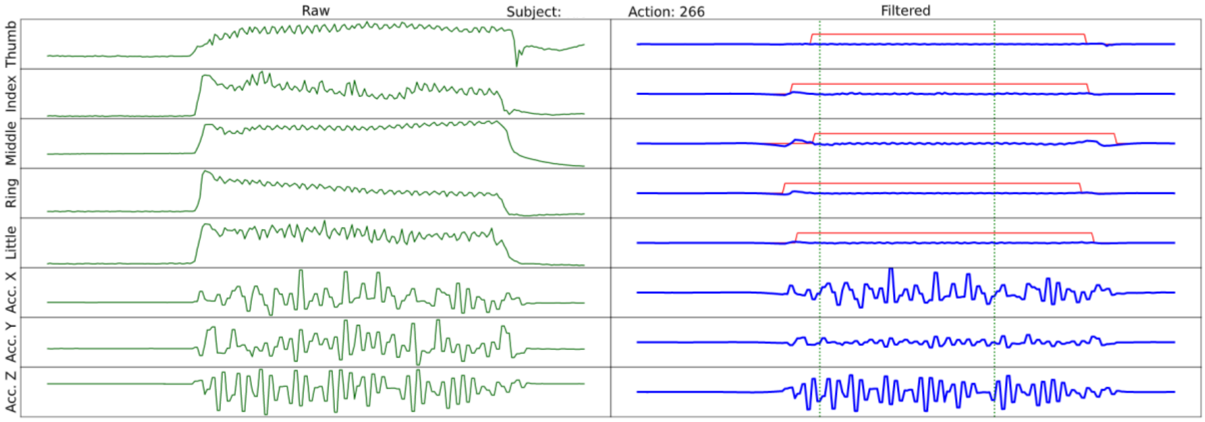

Figure 58. 266 (Rubbing shoulder with high intensity – 10 secs)

271 (Scratch neck by only moving the whole forearm – 10 secs)

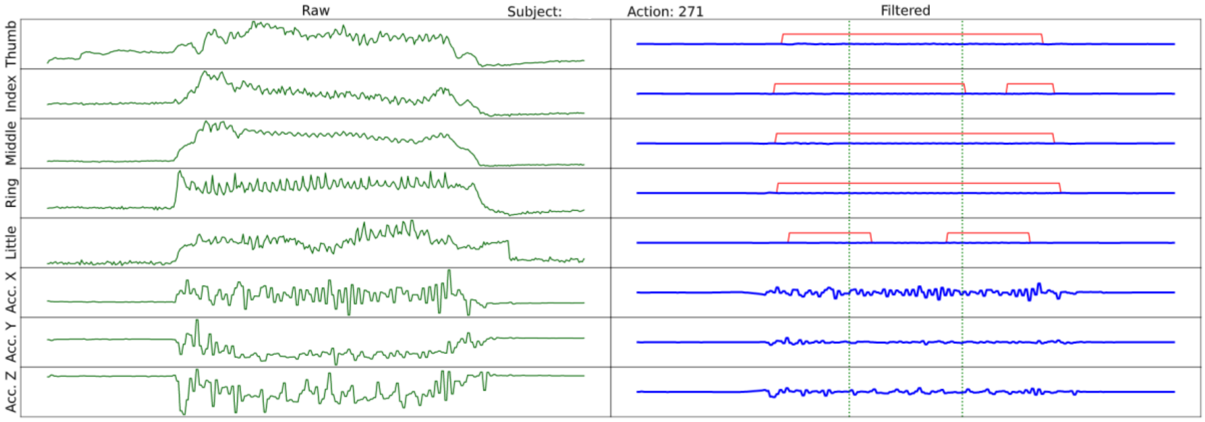

Figure 59. 271 (Scratch neck by only moving the whole forearm – 10 secs)

272 (Scratch neck by only moving the fingers – 10 secs)

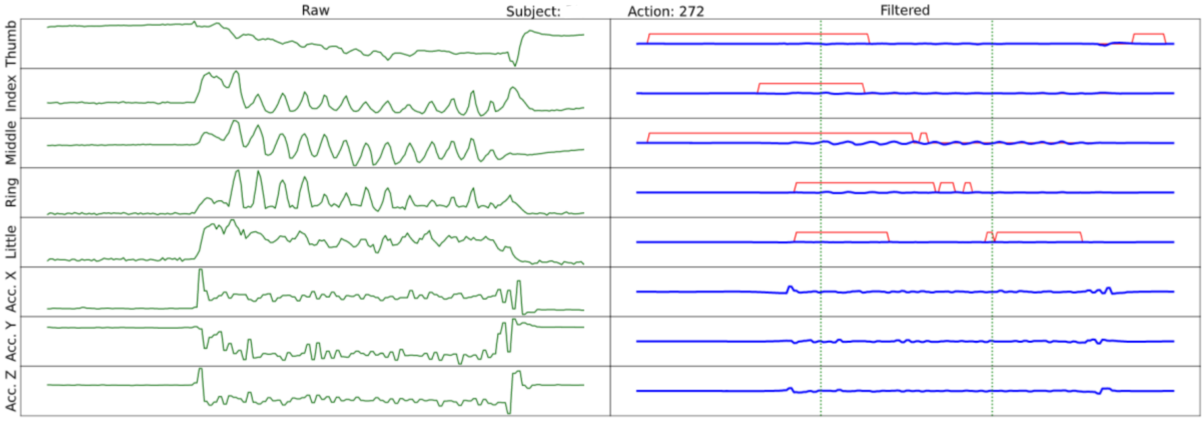

Figure 60. 272 (Scratch neck by only moving the fingers – 10 secs)

273 (Rubbing neck – 10 secs)

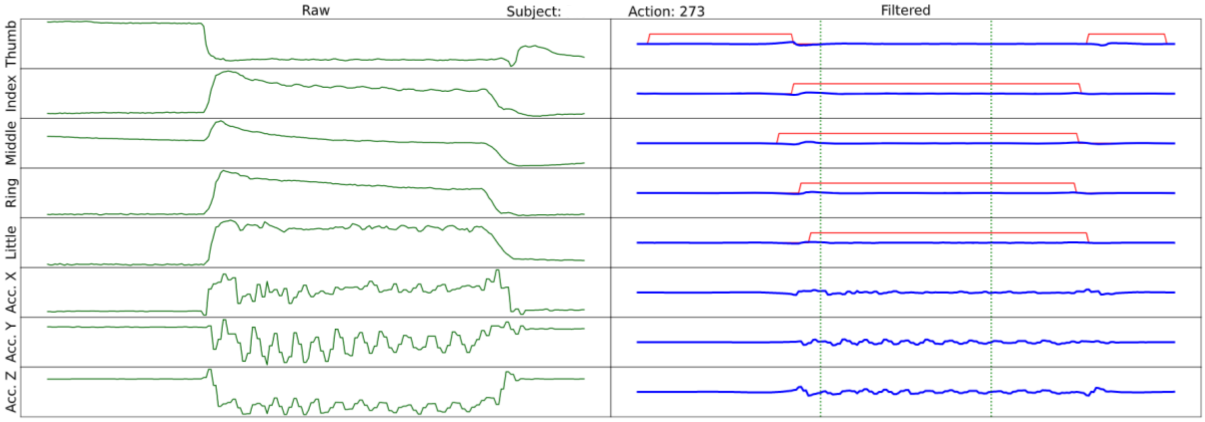

Figure 61. 273 (Rubbing neck – 10 secs)

274 (Scratch neck by only moving the whole forearm with high intensity – 10 secs)

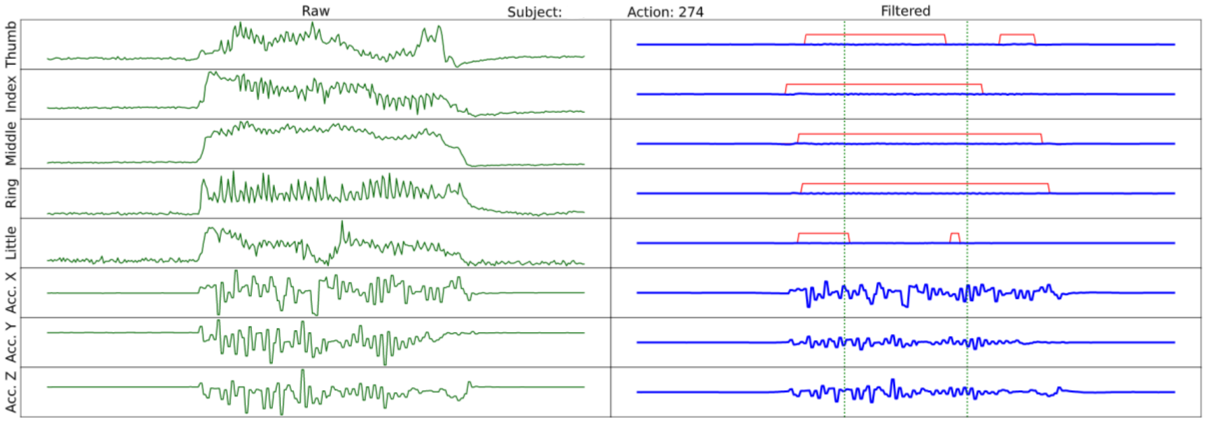

Figure 62. 274 (Scratch neck by only moving the whole forearm with high intensity – 10 secs)

275 (Scratch neck by only moving the fingers with high intensity – 10 secs)

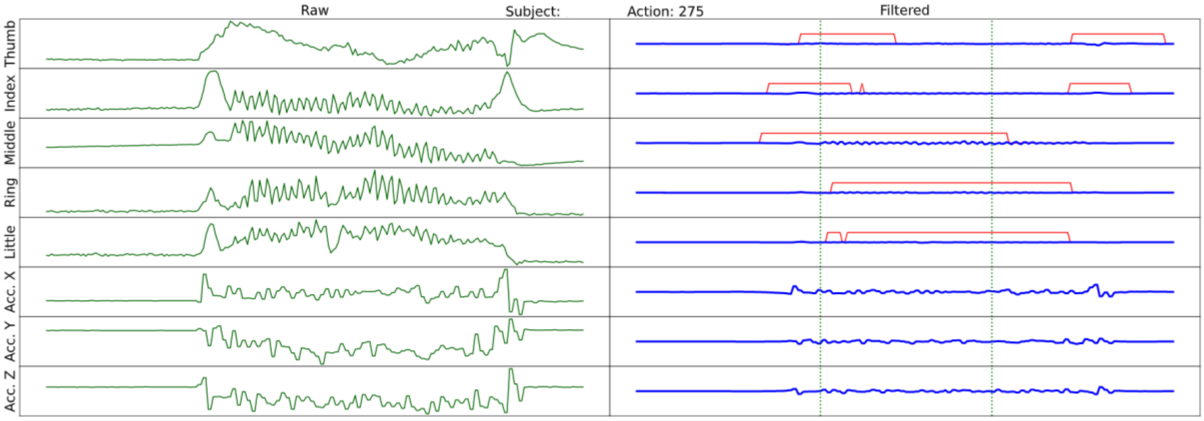

Figure 63. 275 (Scratch neck by only moving the fingers with high intensity – 10 secs)

276 (Rubbing neck with high intensity – 10 secs)

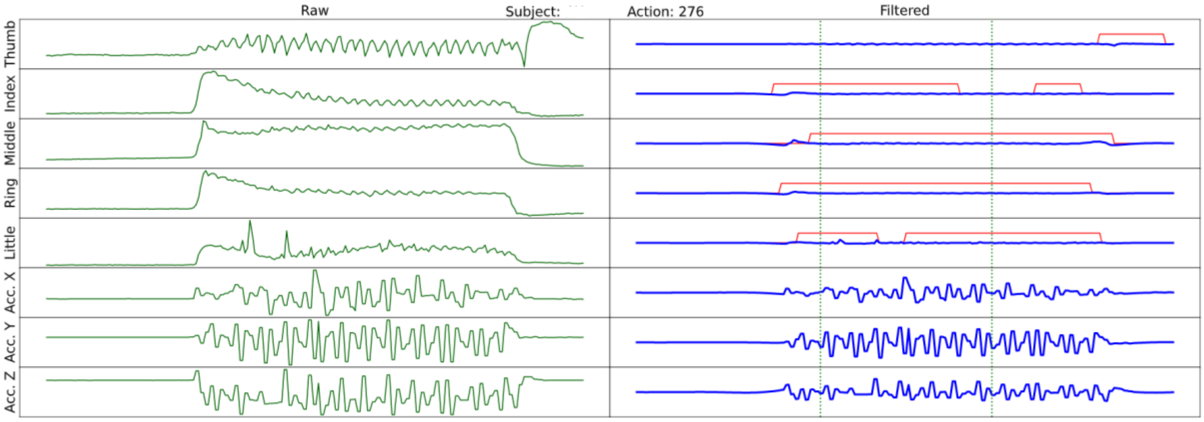

Figure 64. 276 (Rubbing neck with high intensity – 10 secs)

281 (Scratch cheek by only moving the whole forearm – 10 secs)

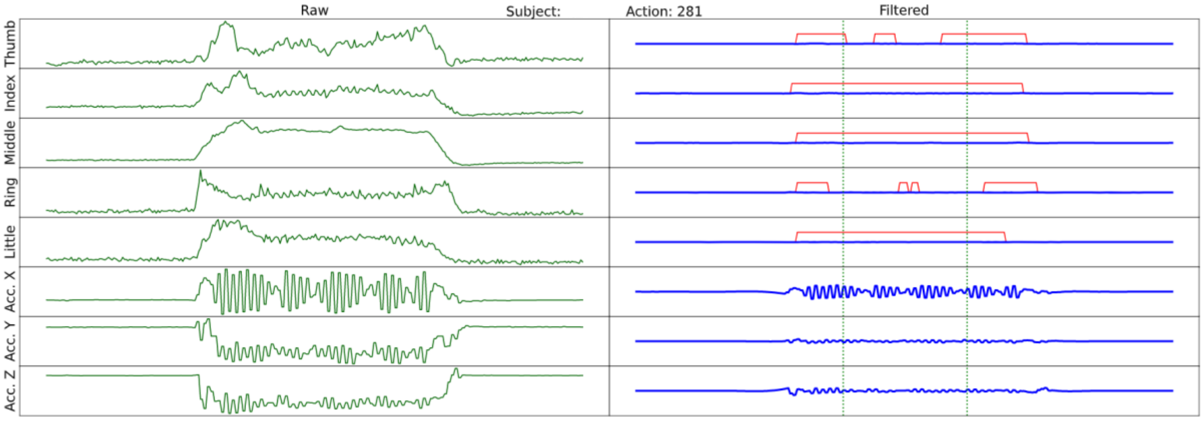

Figure 65. 281 (Scratch cheek by only moving the whole forearm – 10 secs)

282 (Scratch cheek by only moving the fingers – 10 secs)

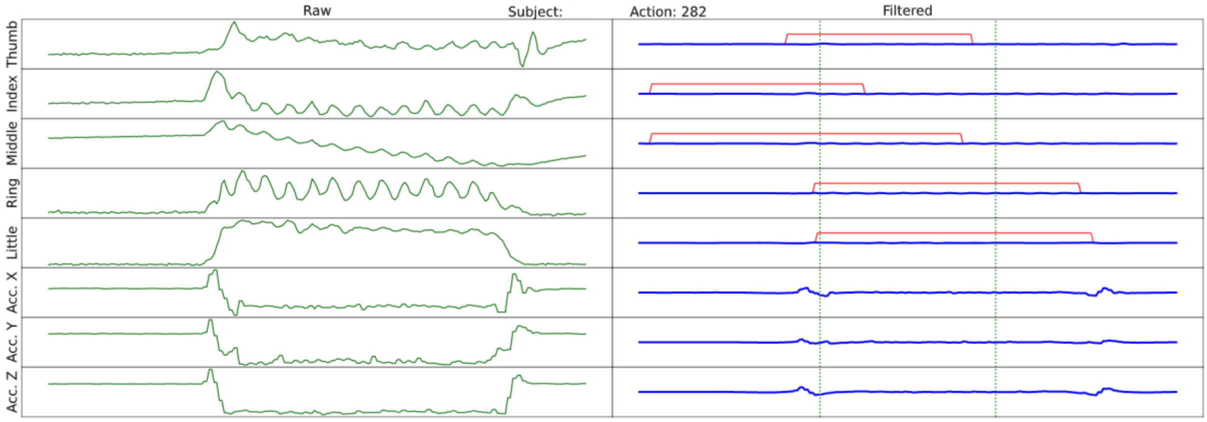

Figure 66. 282 (Scratch cheek by only moving the fingers – 10 secs)

283 (Rubbing cheek – 10 secs)

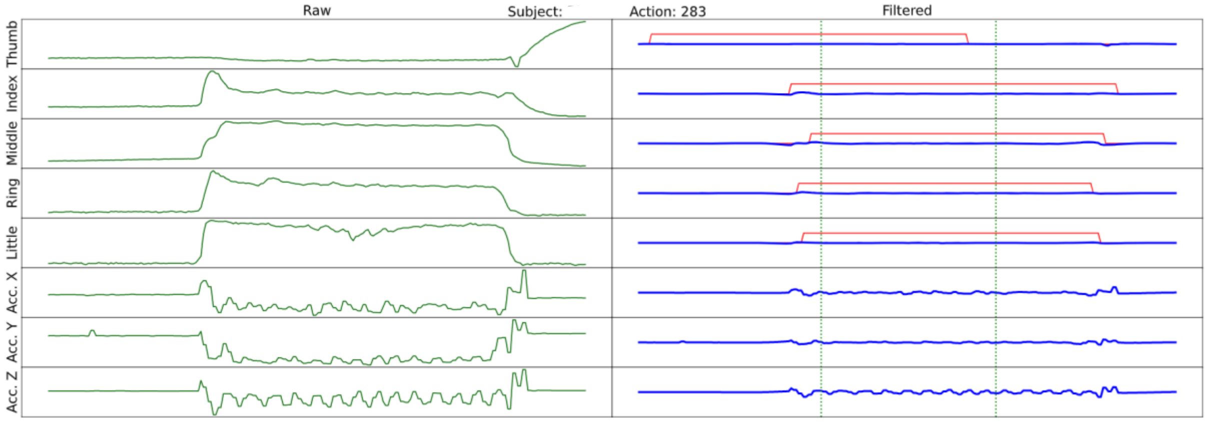

Figure 67. 283 (Rubbing cheek – 10 secs)

284 (Scratch cheek by only moving the whole forearm with high intensity – 10 secs)

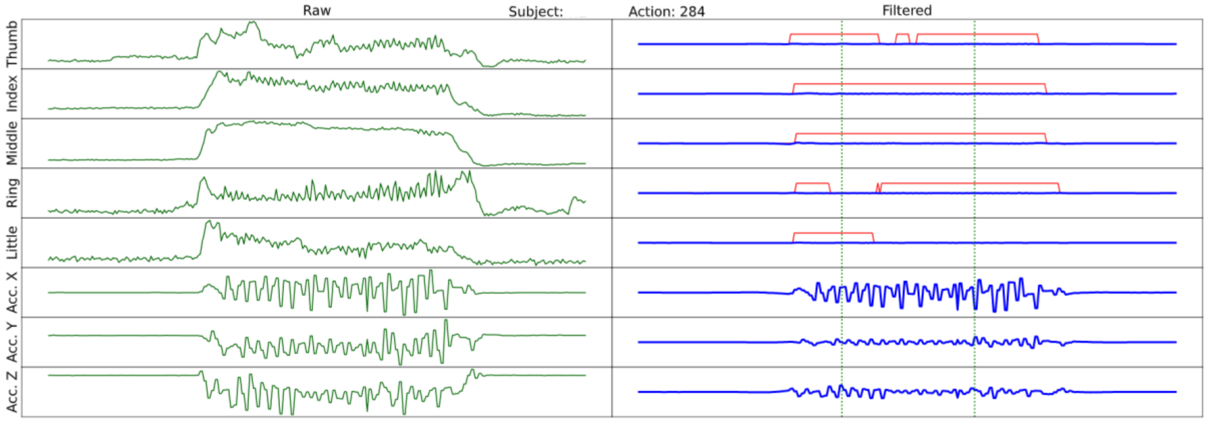

Figure 68. 284 (Scratch cheek by only moving the whole forearm with high intensity – 10 secs)

285 (Scratch cheek by only moving the fingers with high intensity – 10 secs)

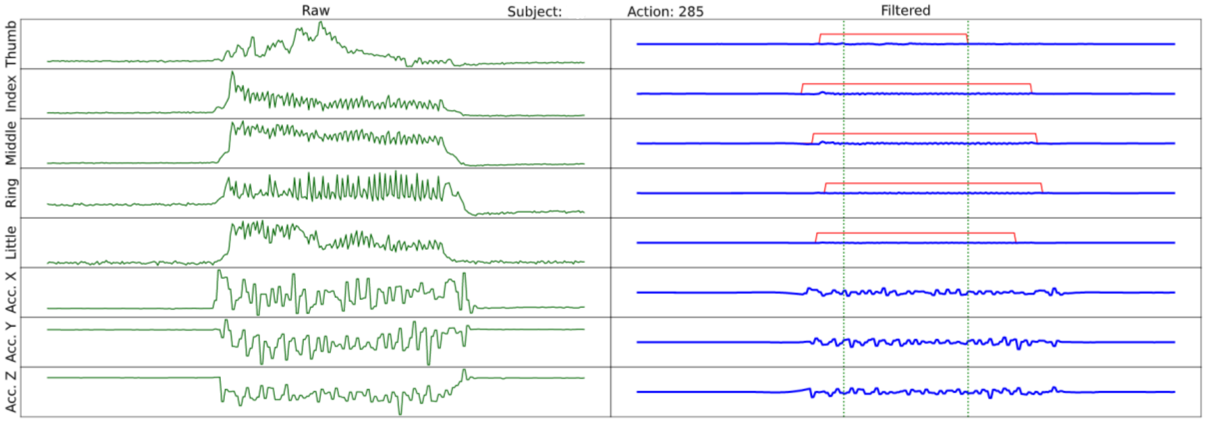

Figure 69. 285 (Scratch cheek by only moving the fingers with high intensity – 10 secs)

286 (Rubbing cheek with high intensity – 10 secs)

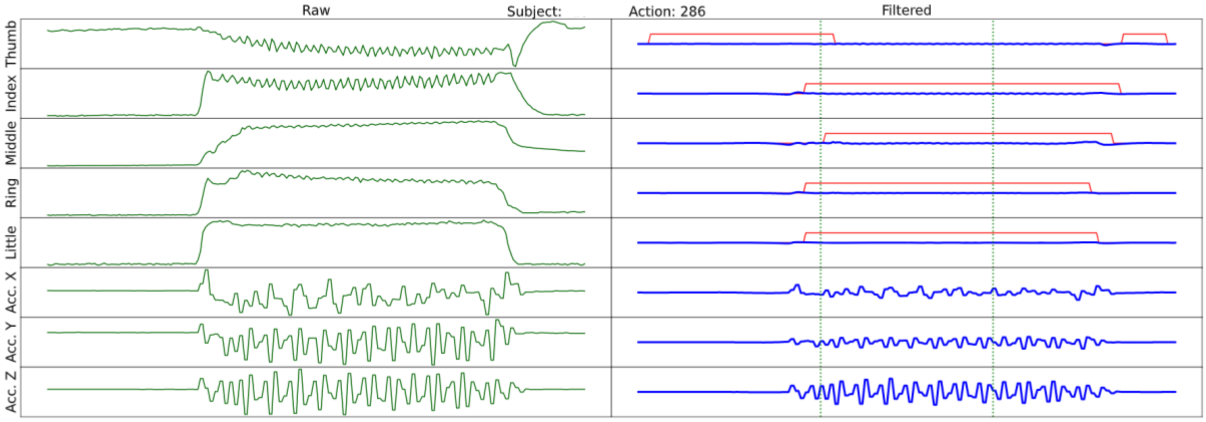

Figure 70. 286 (Rubbing cheek with high intensity – 10 secs)

291 (Scratch top of head by only moving the whole forearm – 10 secs)

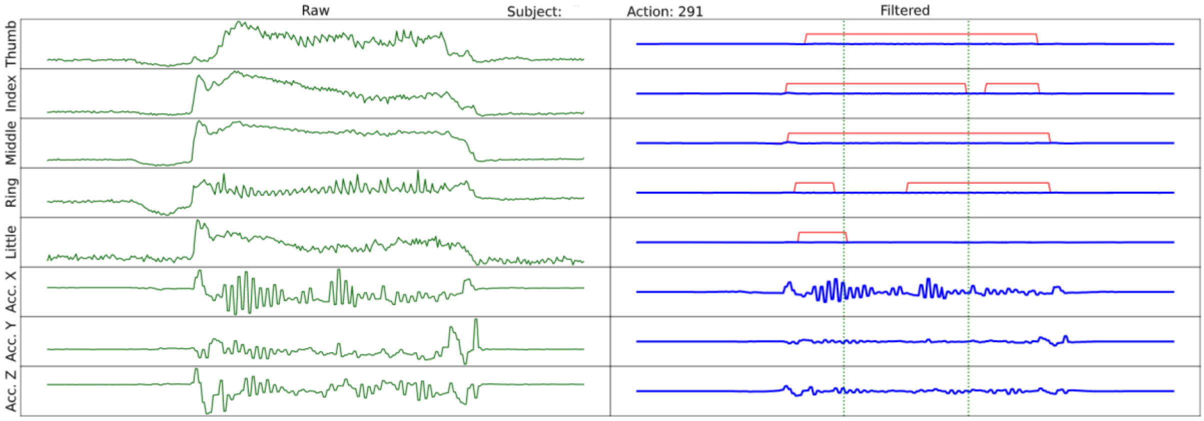

Figure 71. 291 (Scratch top of head by only moving the whole forearm – 10 secs)

292 (Scratch top of head by only moving the fingers – 10 secs)

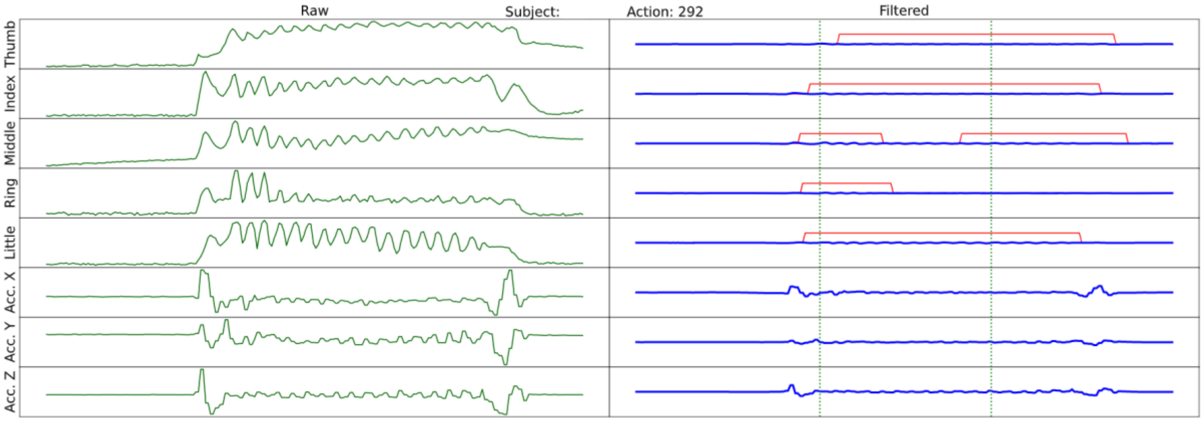

Figure 72. 292 (Scratch top of head by only moving the fingers – 10 secs)

293 (Rubbing top of head – 10 secs)

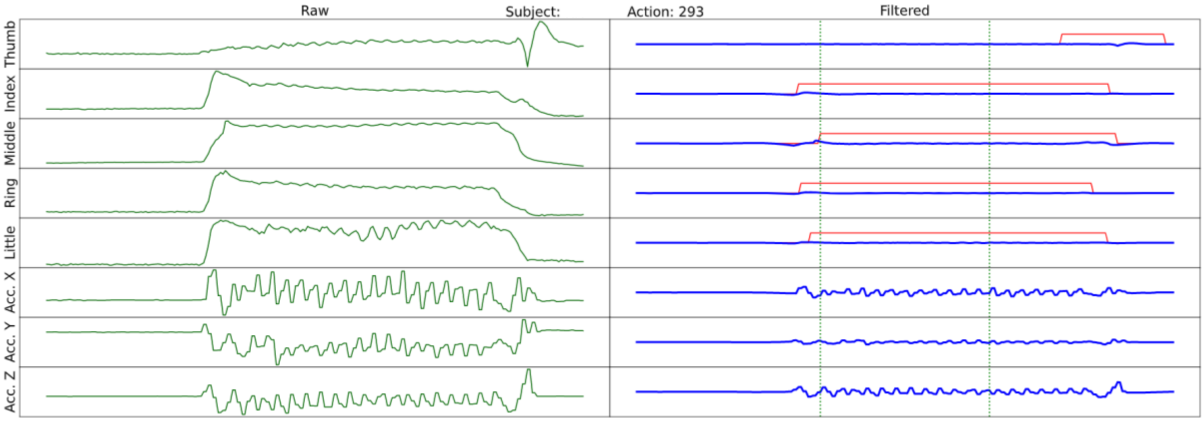

Figure 73. 293 (Rubbing top of head – 10 secs)

294 (Scratch top of head by only moving the whole forearm with high intensity – 10 secs)

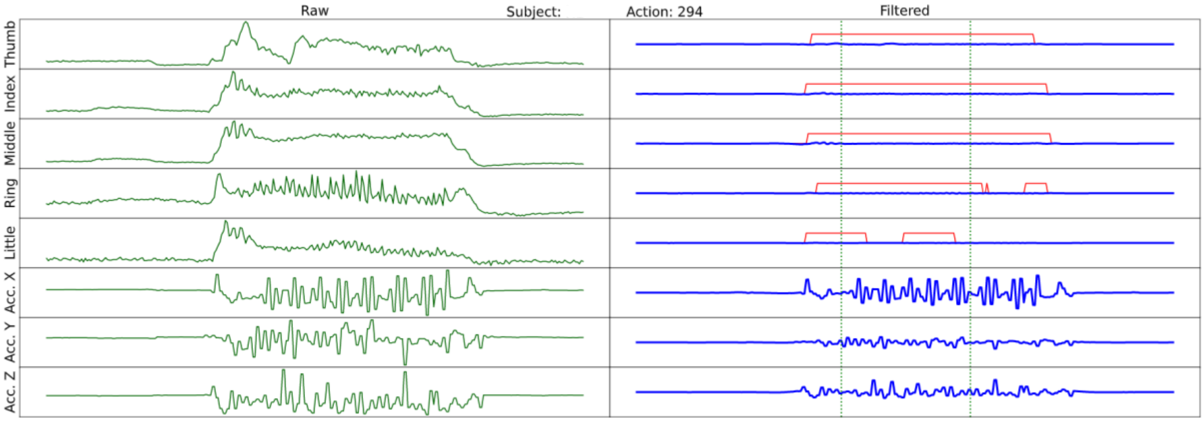

Figure 74. 294 (Scratch top of head by only moving the whole forearm with high intensity – 10 secs)

295 (Scratch top of head by only moving the fingers with high intensity – 10 secs)

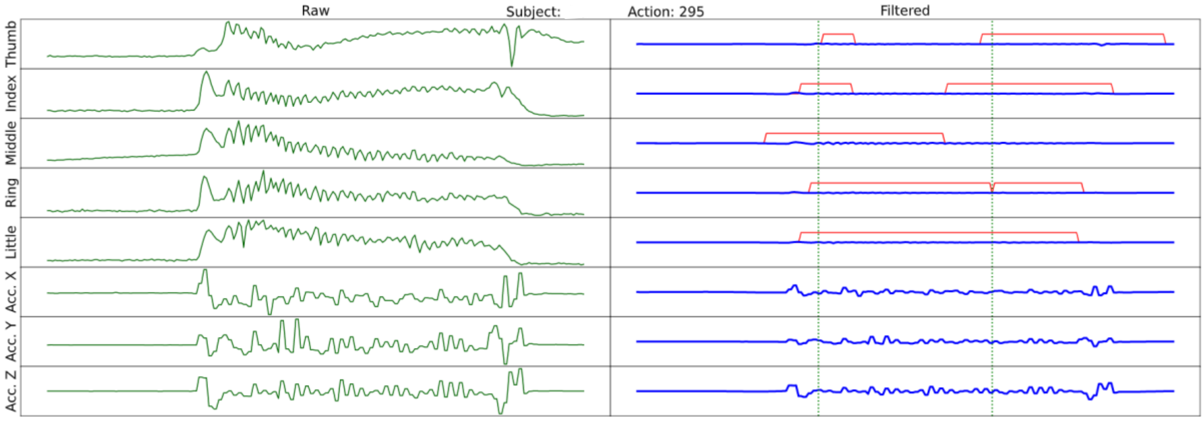

Figure 75. 295 (Scratch top of head by only moving the fingers with high intensity – 10 secs)

296 (Rubbing top of head with high intensity – 10 secs)

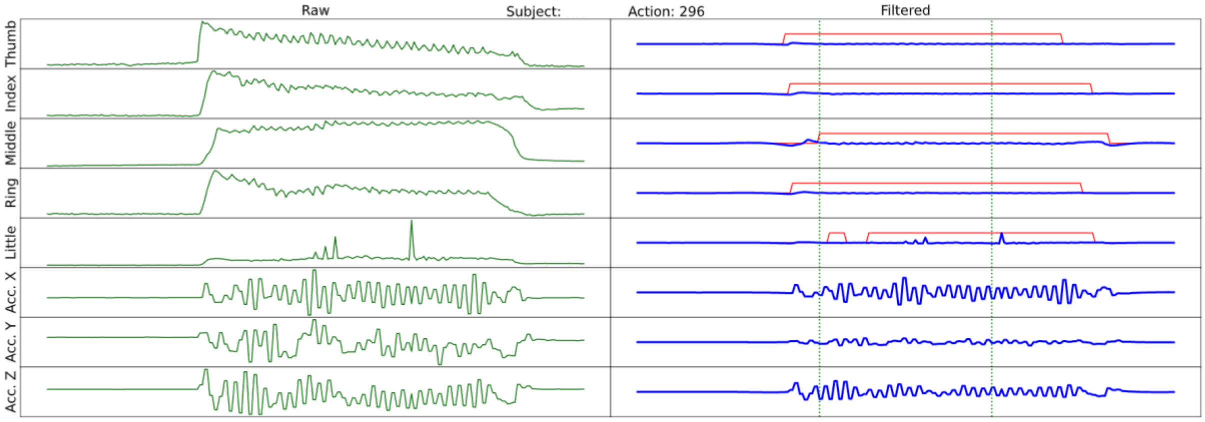

Figure 76. 296 (Rubbing top of head with high intensity – 10 secs)

301 (Scratch calf by only moving the whole forearm – 10 secs)

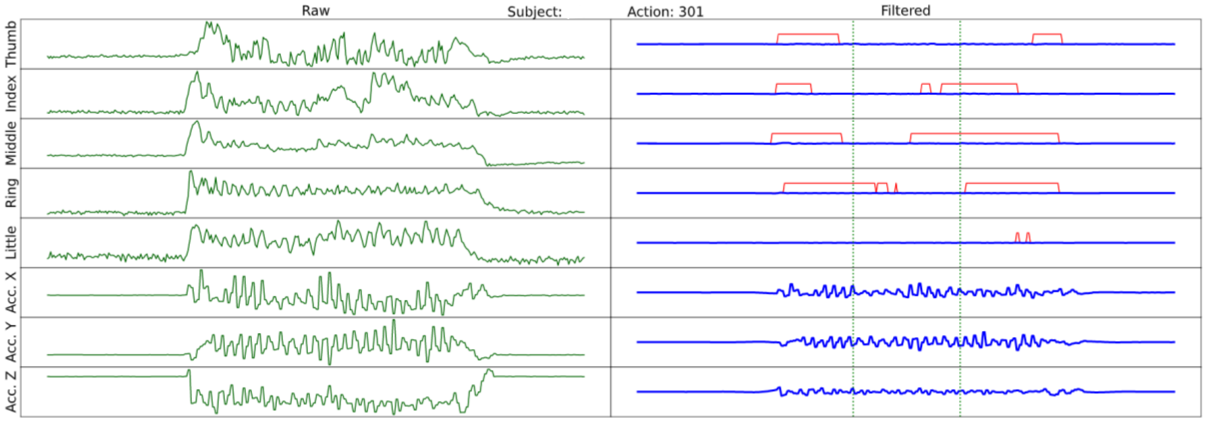

Figure 77. 301 (Scratch calf by only moving the whole forearm – 10 secs)

302 (Scratch calf by only moving the fingers – 10 secs)

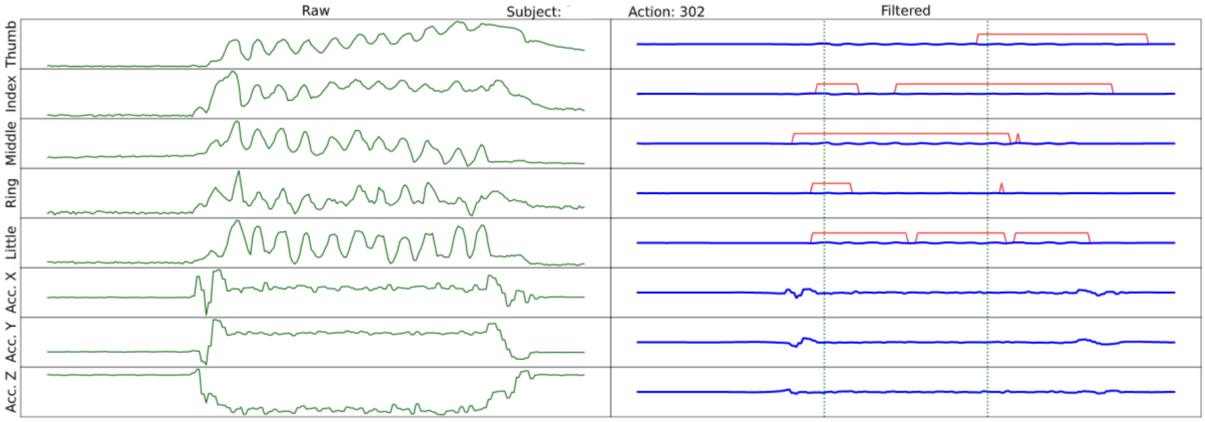

Figure 78. 302 (Scratch calf by only moving the fingers – 10 secs)

303 (Rubbing calf – 10 secs)

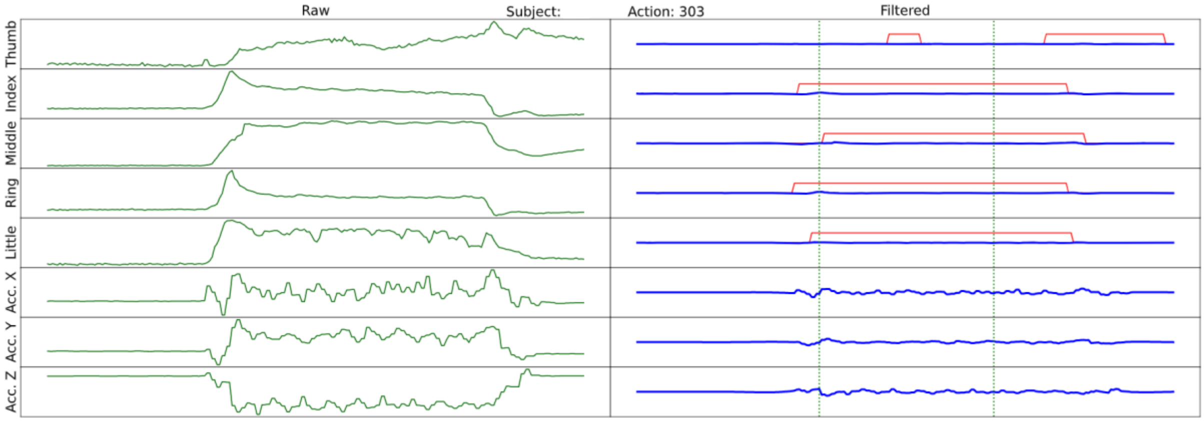

Figure 79. 303 (Rubbing calf – 10 secs)

304 (Scratch calf by only moving the whole forearm with high intensity – 10 secs)

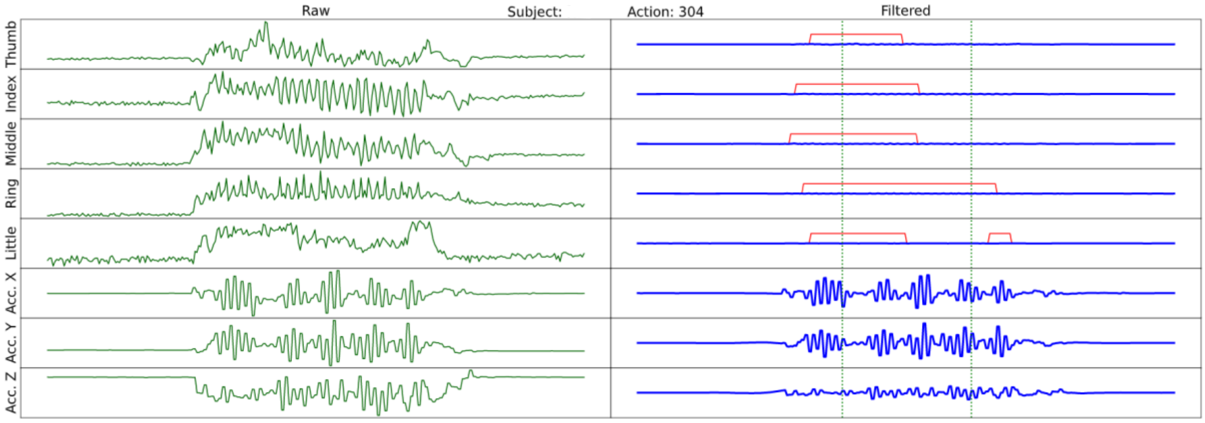

Figure 80. 304 (Scratch calf by only moving the whole forearm with high intensity – 10 secs)

305 (Scratch calf by only moving the fingers with high intensity – 10 secs)

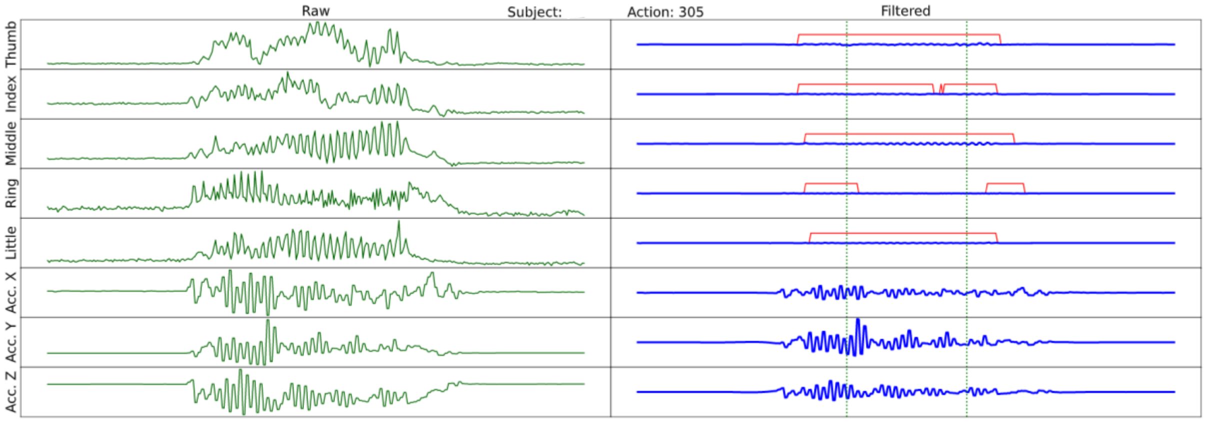

Figure 81. 305 (Scratch calf by only moving the fingers with high intensity – 10 secs)

306 (Rubbing calf with high intensity – 10 secs)

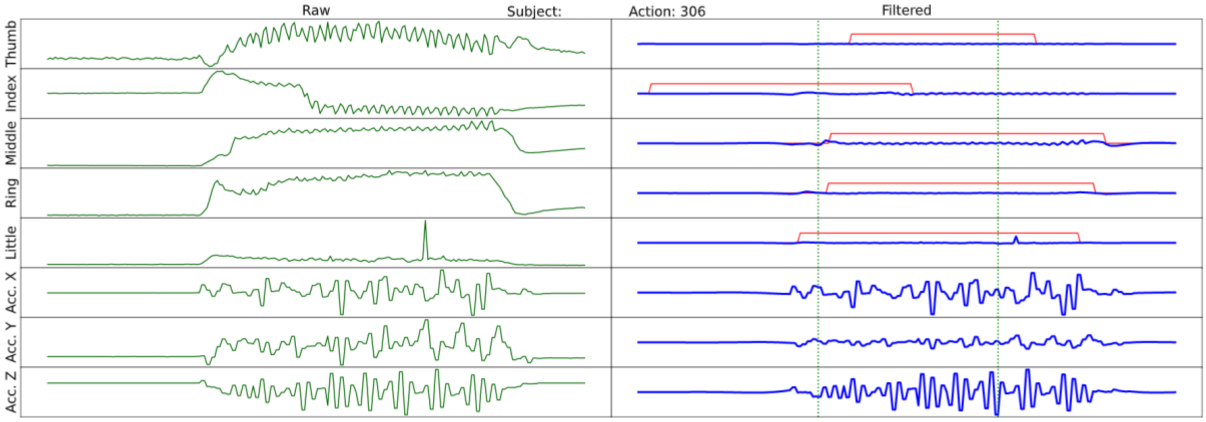

Figure 82. 306 (Rubbing calf with high intensity – 10 secs)

311 (Scratch knee by only moving the whole forearm – 10 secs)

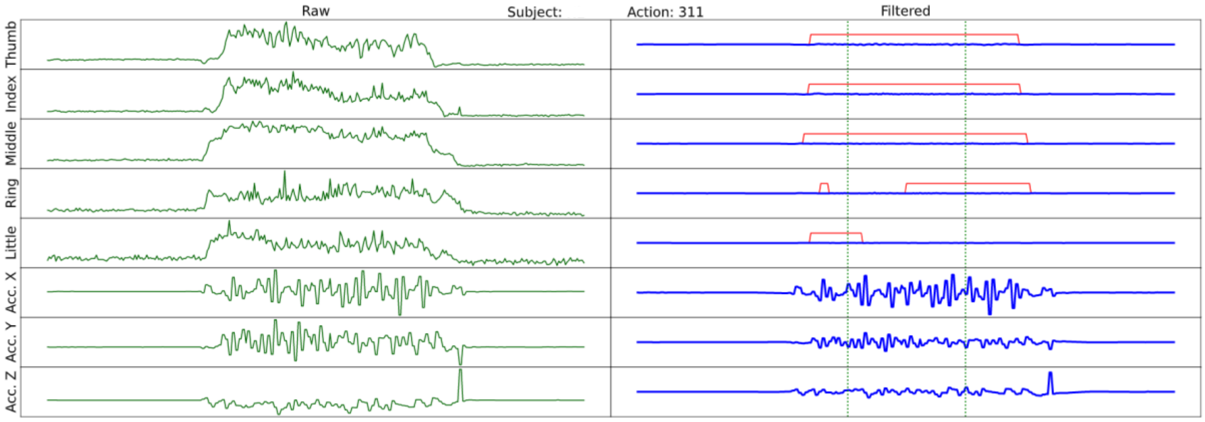

Figure 83. 311 (Scratch knee by only moving the whole forearm – 10 secs)

312 (Scratch knee by only moving the fingers – 10 secs)

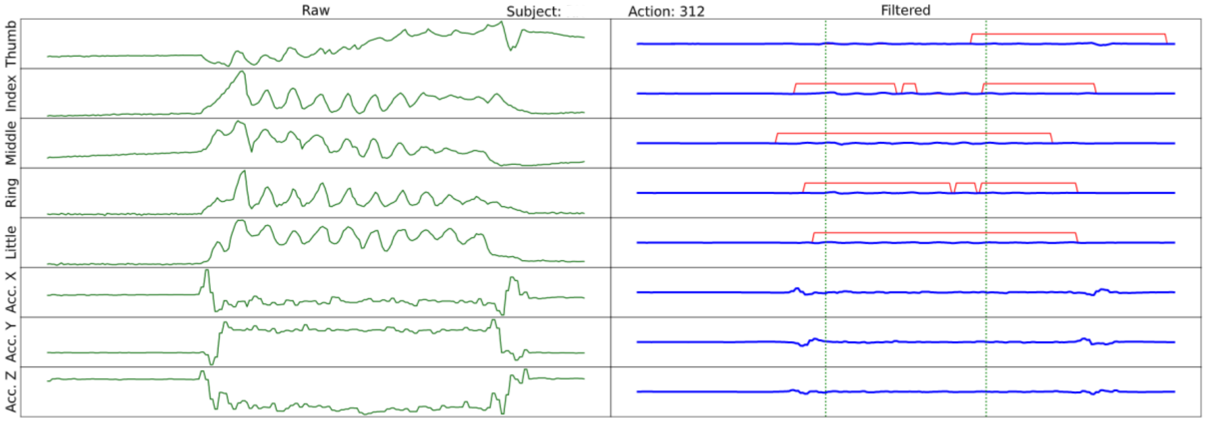

Figure 84. 312 (Scratch knee by only moving the fingers – 10 secs)

313 (Rubbing knee – 10 secs)

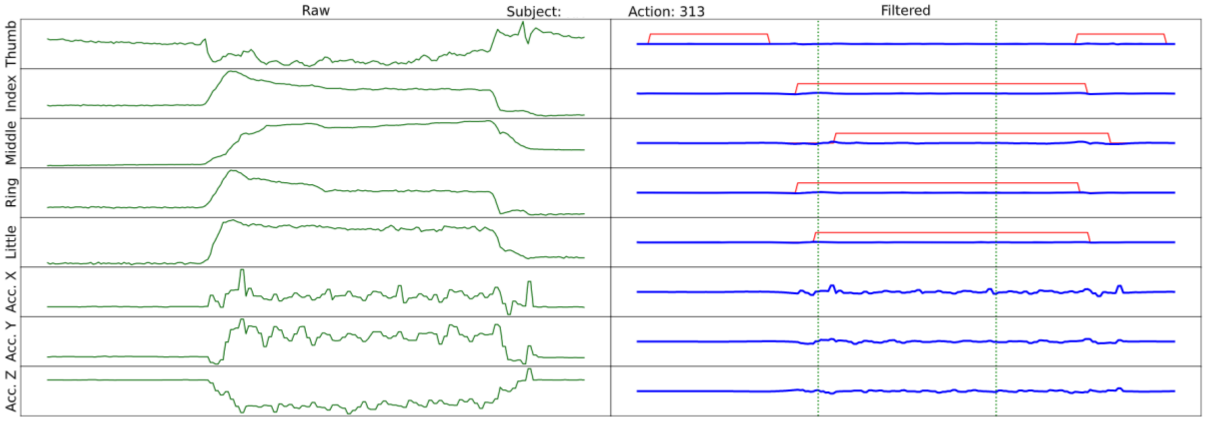

Figure 85. 313 (Rubbing knee – 10 secs)

314 (Scratch knee by only moving the whole forearm with high intensity – 10 secs)

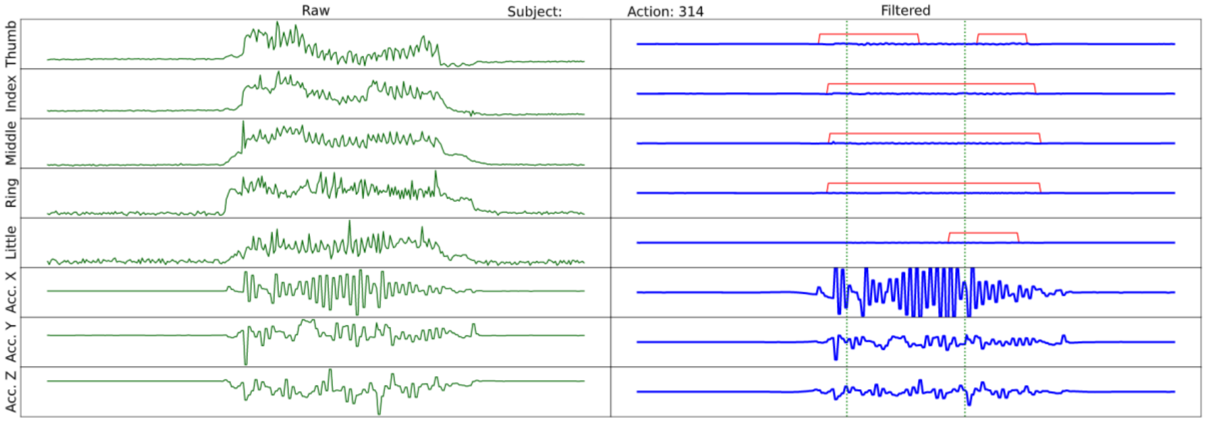

Figure 86. 314 (Scratch knee by only moving the whole forearm with high intensity – 10 secs)

315 (Scratch knee by only moving the fingers with high intensity – 10 secs)

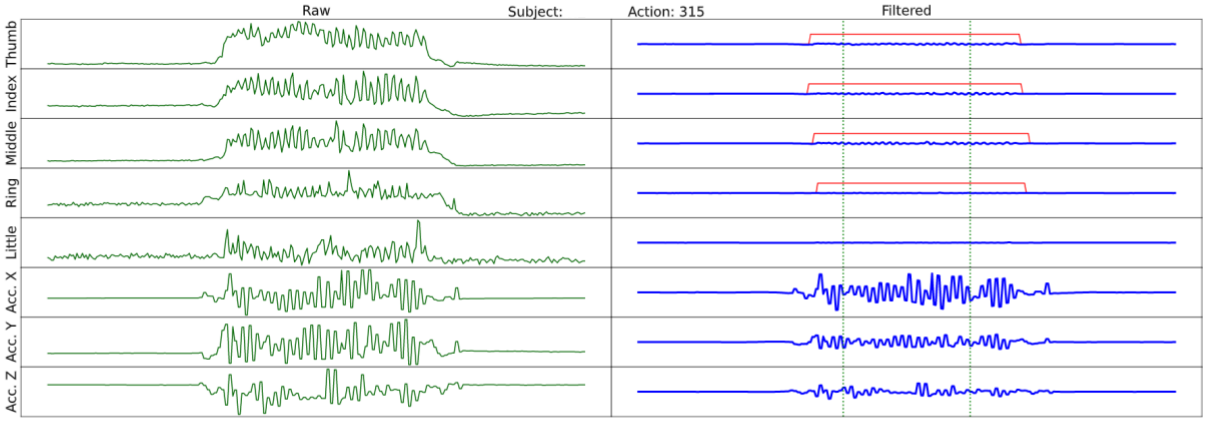

Figure 87. 315 (Scratch knee by only moving the fingers with high intensity – 10 secs)

316 (Rubbing knee with high intensity – 10 secs)

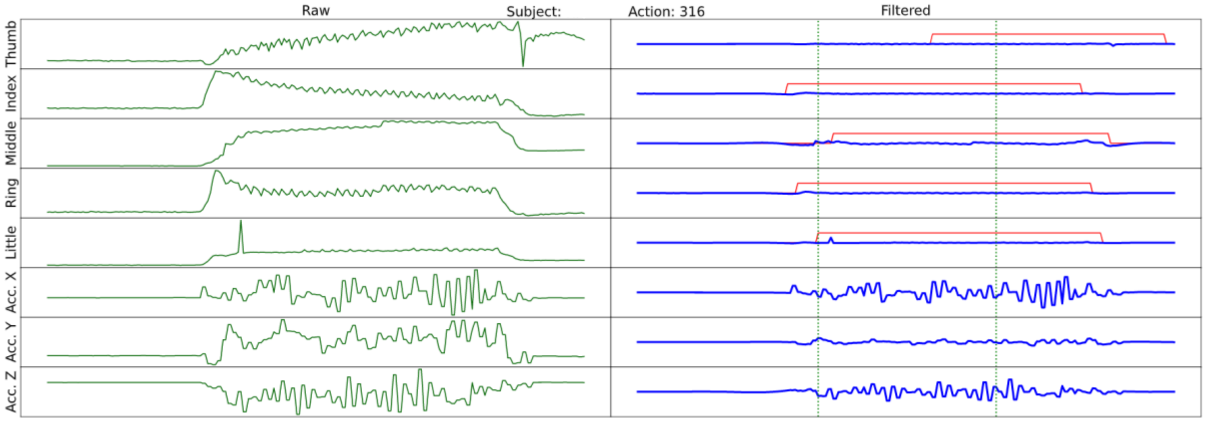

Figure 88. 316 (Rubbing knee with high intensity – 10 secs)

321 (Scratch top of thigh by only moving the whole forearm – 10 secs)

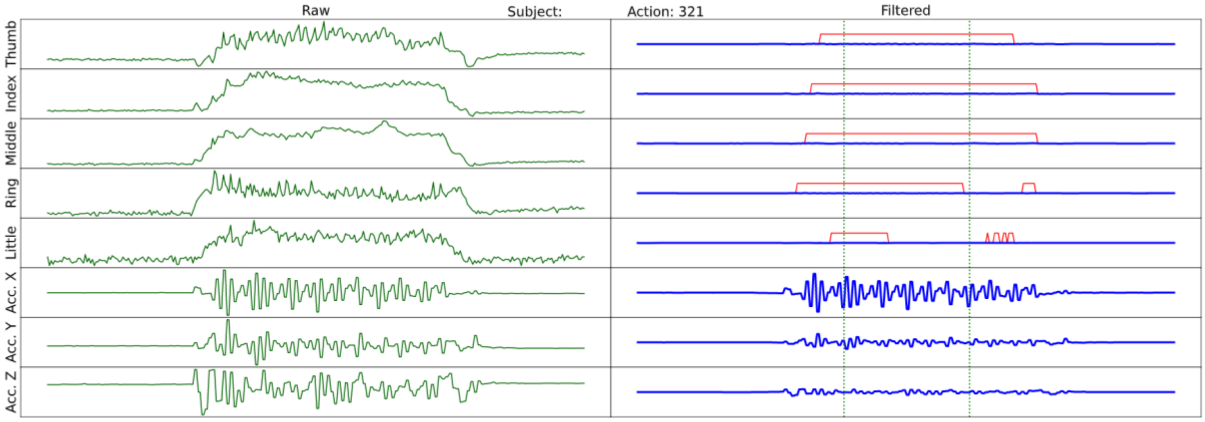

Figure 89. 321 (Scratch top of thigh by only moving the whole forearm – 10 secs)

322 (Scratch top of thigh by only moving the fingers – 10 secs)

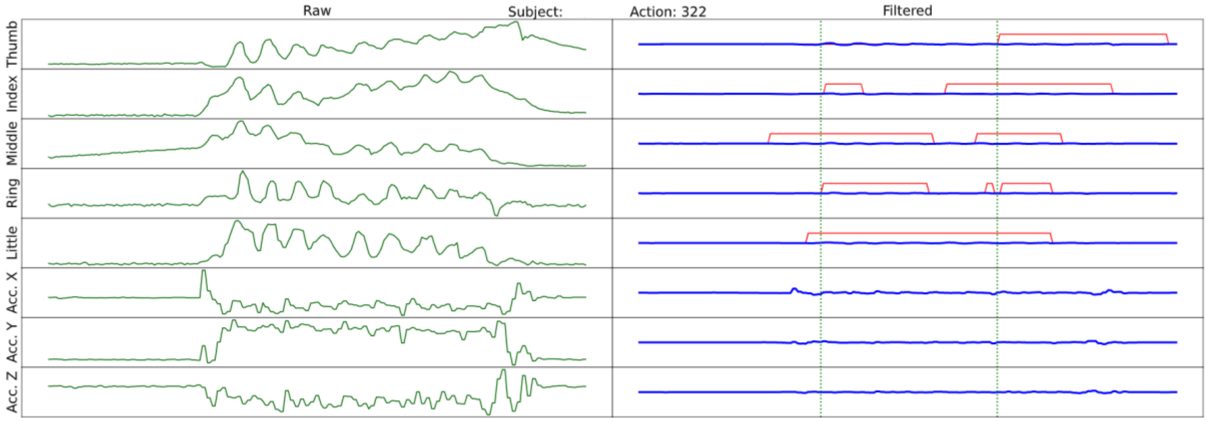

Figure 90. 322 (Scratch top of thigh by only moving the fingers – 10 secs)

323 (Rubbing top of thigh – 10 secs)

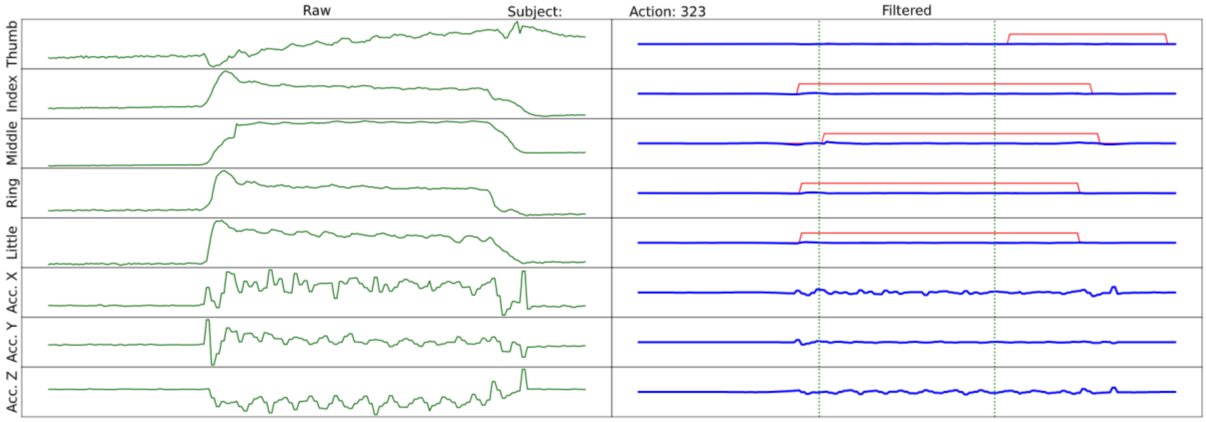

Figure 91. 323 (Rubbing top of thigh – 10 secs)

324 (Scratch top of thigh by only moving the whole forearm with high intensity – 10 secs)

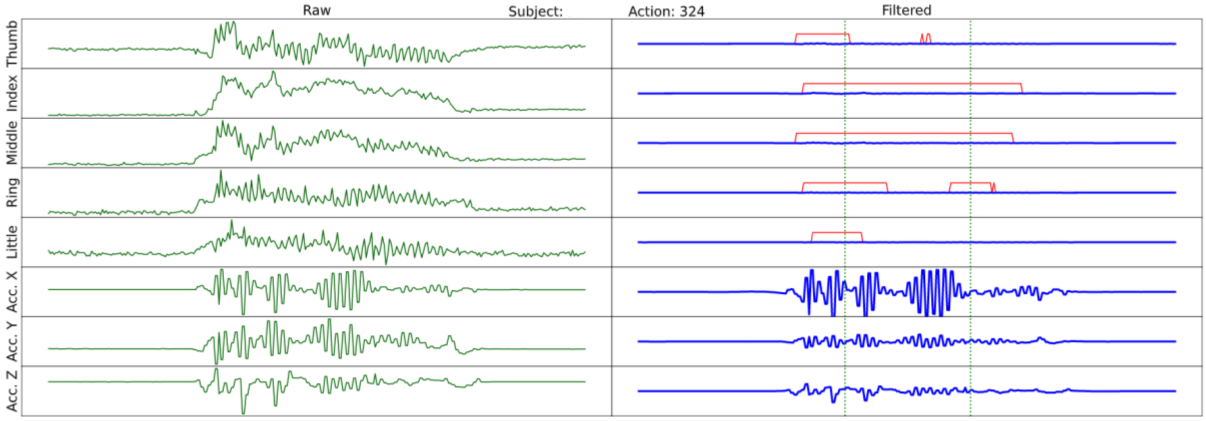

Figure 92. 324 (Scratch top of thigh by only moving the whole forearm with high intensity – 10 secs)

325 (Scratch top of thigh by only moving the fingers with high intensity – 10 secs)

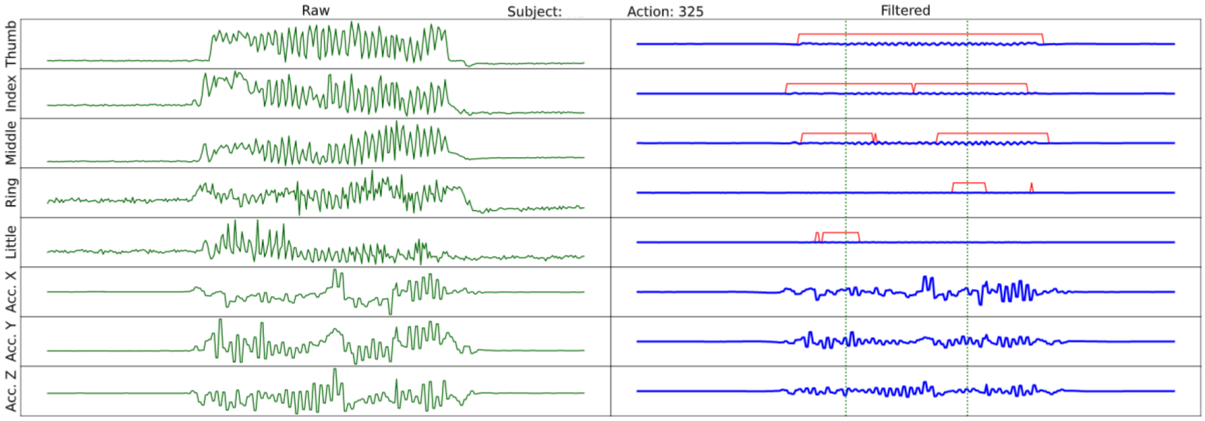

Figure 93. 325 (Scratch top of thigh by only moving the fingers with high intensity – 10 secs)

326 (Rubbing top of thigh with high intensity – 10 secs)

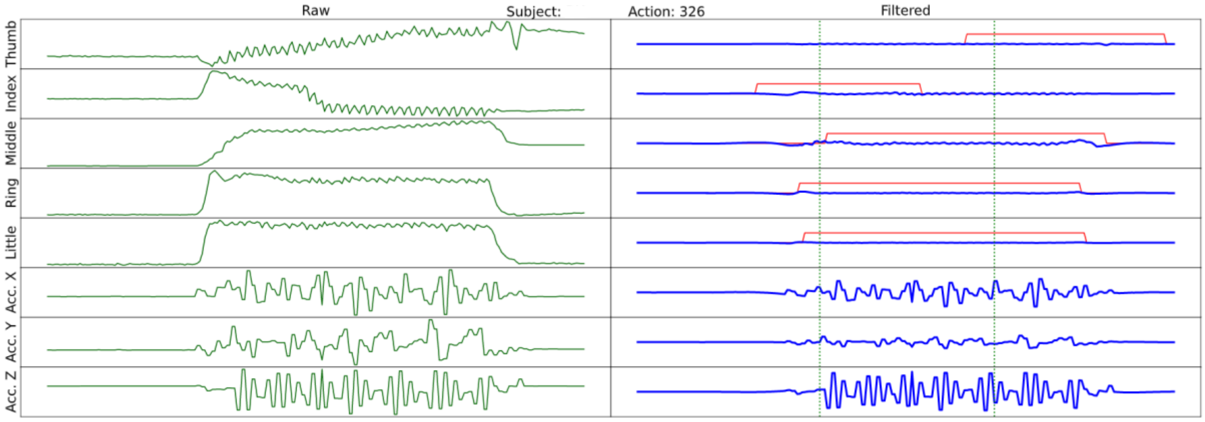

Figure 94. 326 (Rubbing top of thigh with high intensity – 10 secs)

331 (Scratch abdomen by only moving the whole forearm – 10 secs)

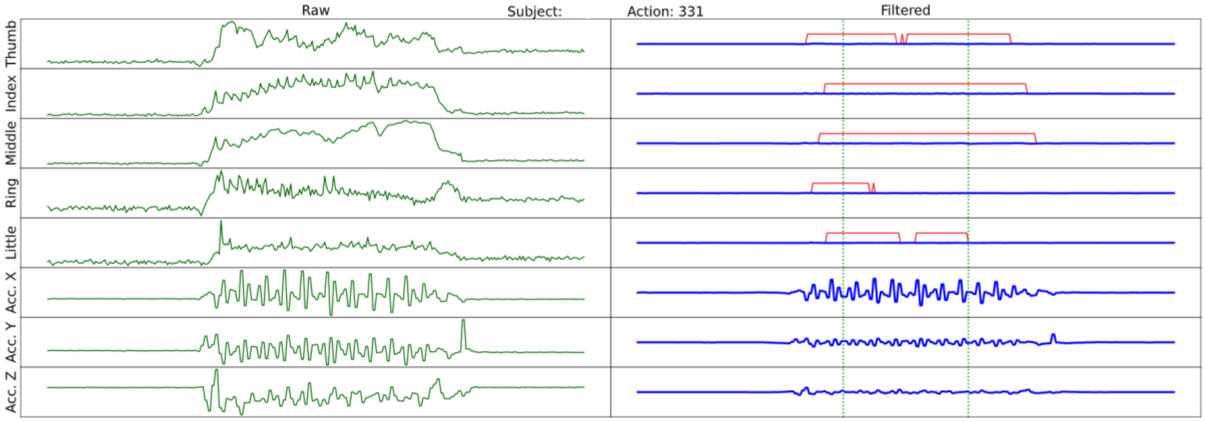

Figure 95. 331 (Scratch abdomen by only moving the whole forearm – 10 secs)

332 (Scratch abdomen by only moving the fingers – 10 secs)

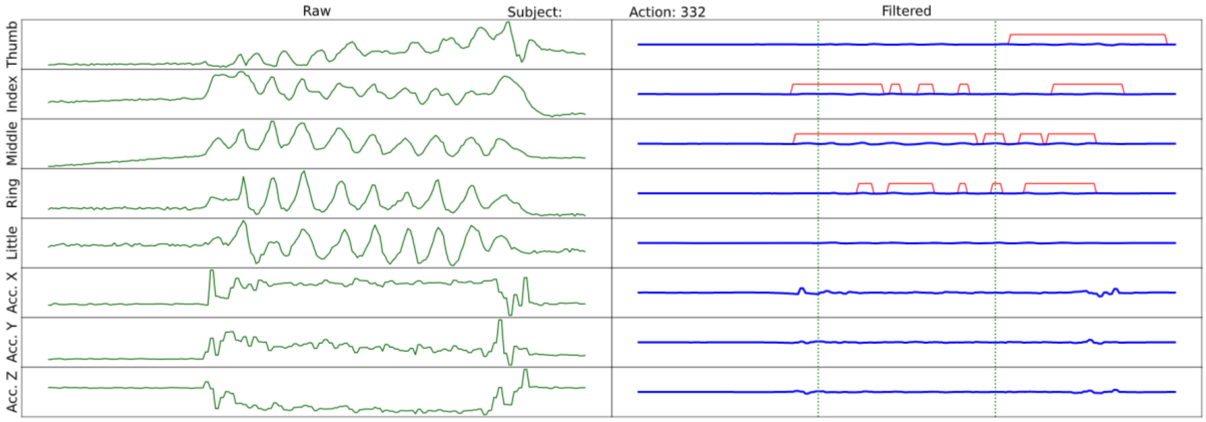

Figure 96. 332 (Scratch abdomen by only moving the fingers – 10 secs)

333 (Rubbing abdomen – 10 secs)

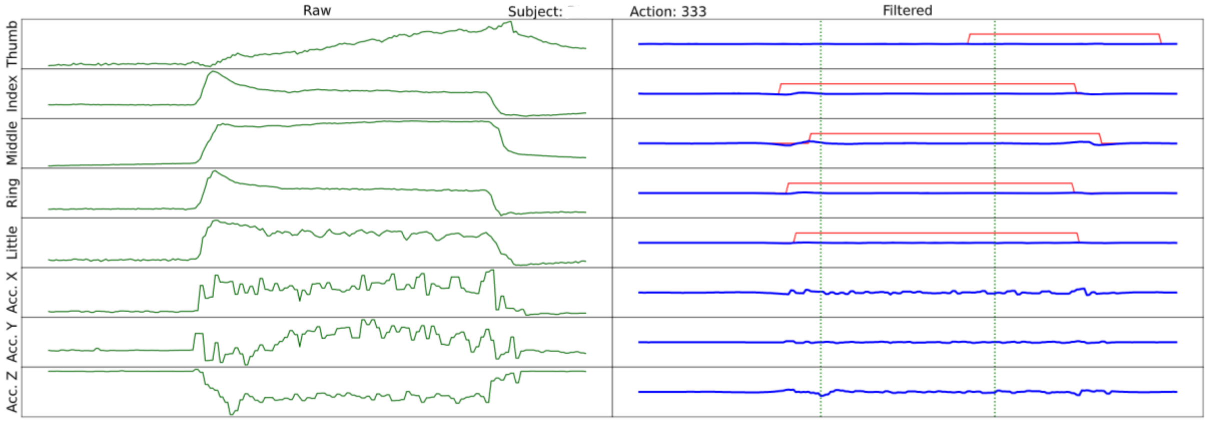

Figure 97. 333 (Rubbing abdomen – 10 secs)

334 (Scratch abdomen by only moving the whole forearm with high intensity – 10 secs)

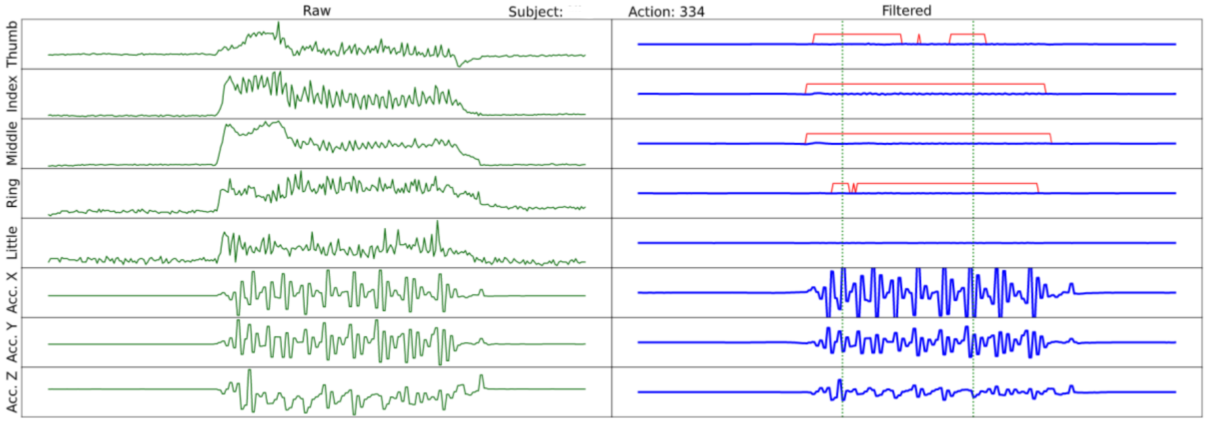

Figure 98. 334 (Scratch abdomen by only moving the whole forearm with high intensity – 10 secs)

335 (Scratch abdomen by only moving the fingers with high intensity – 10 secs)

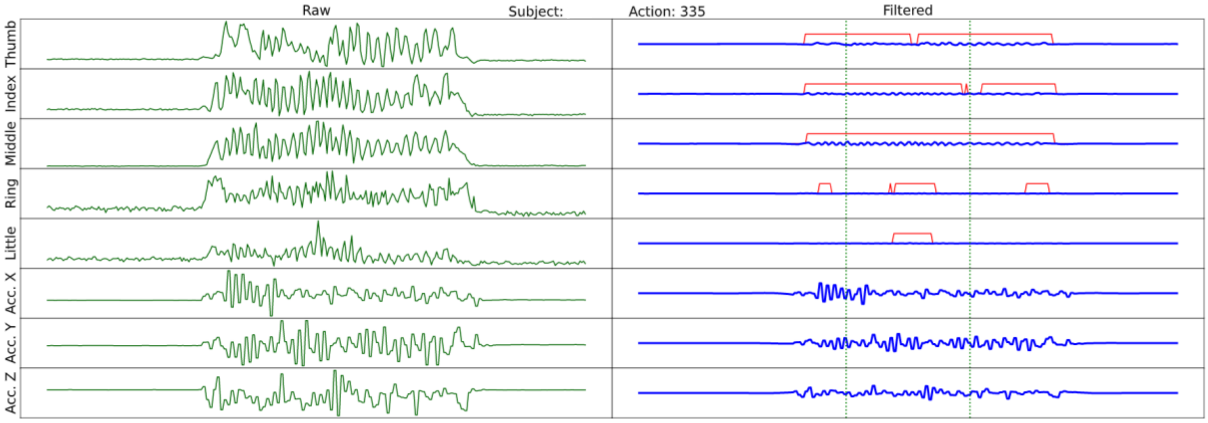

Figure 99. 335 (Scratch abdomen by only moving the fingers with high intensity – 10 secs)

336 (Rubbing abdomen with high intensity – 10 secs)

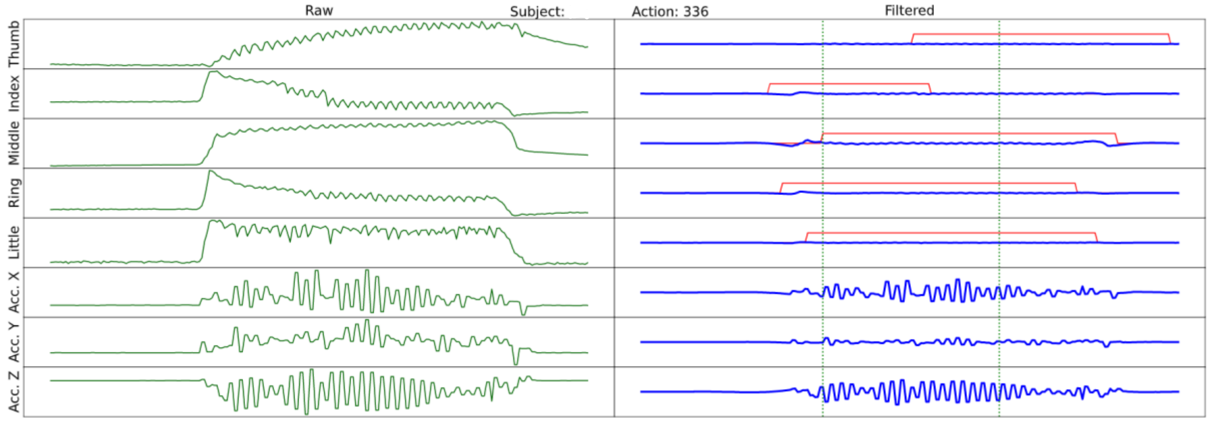

Figure 100. 336 (Rubbing abdomen with high intensity – 10 secs)
